# Supplementary material for: Using genome and transcriptome data from African-ancestry female participants to identify putative breast cancer susceptibility genes
Source: Nat Commun. 2024 May 2;15:3718. doi: 10.1038/s41467-024-47650-5 (PMC11065893; doi:10.1038/s41467-024-47650-5)

Using genome and transcriptome data from African-ancestry females to identify putative breast cancer susceptibility genes

Jie Ping[1], Guochong Jia[1], Qiuyin Cai[1], Xingyi Guo[1], Ran Tao[2,3], Christine Ambrosone[4], Dezheng Huo[5], Stefan Ambs[6], Mollie E. Barnard[7], Yu Chen[8], Montserrat Garcia-Closas[9], Jian Gu[10], Jennifer J. Hu[11], Esther M. John[12], Christopher I. Li[13], Katherine Nathanson[14,15], Barbara Nemesure[16], Olufunmilayo I. Olopade[17], Tuya Pal[18], Michael F. Press[19], Maureen Sanderson[20], Dale P. Sandler[21], Toshio Yoshimatsu[5], Prisca O. Adejumo[22], Thomas Ahearn[9], Abenaa M. Brewster[23], Anselm J.M. Hennis[24,25], Timothy Makumbi[26], Paul Ndom[27], Katie M. O'Brien[21], Andrew F. Olshan[28], Mojisola M. Oluwasanu[29], Sonya Reid[30], Song Yao[4], Ebonee N. Butler[28], Maosheng Huang[10], Atara Ntekim[31], Bingshan Li[32], Melissa A. Troester[28], Julie R. Palmer[7], Christopher A. Haiman[33], Jirong Long[1], Wei Zheng[1*]

SUPPLEMENTARY MATERIALS

Study Participants of the African-ancestry Breast Cancer Genetic (AABCG) consortium (in no particular order of ranking)

<u>Southern Community Cohort Study (SCCS):</u> The SCCS is a prospective cohort study focused on the recruitment of a low-income, predominantly African American population from a 12-state area of southeastern U.S.[1,2] Approximately 86,000 study participants aged 40-79 years were recruited between 2002 and 2009. About 32,500 of the SCCS participants were African American females. An in-person interview was completed at enrollment. Participants were asked to donate a 20-ml blood sample, and a buccal cell or saliva specimen was accepted if the subject did not wish to donate blood. To obtain follow-up data on cancer development, procedures for data linkage, processing, and quality control were established with the 12-state cancer registries covering the SCCS catchment area (Alabama, Arkansas, Florida, Georgia, Kentucky, Louisiana, Mississippi, North Carolina, South Carolina, Tennessee, Virginia, and West Virginia).

Nashville Breast Health Study (NBHS): The NBHS is a population-based case-control study conducted in the Nashville metropolitan area.[3] Participants were recruited between 2001 and 2011. Eligible cases were females newly diagnosed with primary breast cancer between 25 and 75 years of age and with no prior history of cancer other than nonmelanoma skin cancer. Breast cancer cases (n =2,694) were identified through the Tennessee State Cancer Registry and five major hospitals in Nashville that provide medical care for breast cancer patients. Controls (n =2,384) were identified via random digit dialing of households in the same geographic area as the cases. Saliva samples were collected as a source of genomic DNA for genetic studies of breast cancer.

Southern Tri-State Breast Health Study (STSBHS): The STSBHS is a population-based case-only study conducted in Tennessee, South Carolina, and Georgia.[4] It recruited females with incident invasive breast cancer diagnosed between ages 25 and 75 years from 2012 to 2018, without any prior cancer history other than non-melanoma skin cancer. Breast cancer cases were identified through the Tennessee Cancer Registry, the South Carolina Central Cancer Registry, and the Georgia Comprehensive Cancer Registry.

Black Women: Etiology and Survival of Triple-negative Breast Cancers Study (BEST): The BEST is a case-only study which recruited African-ancestry females who were diagnosed with invasive breast cancer at age ≤50 years between 2009 and 2012 and lived in Florida at the time of their diagnosis.[5] Breast cancer cases were identified through the Florida Cancer Registry. Participants provided a saliva sample through mail for DNA extraction and BRCA testing.

Vanderbilt Biobank (BioVU): The DNA biobank at Vanderbilt University consists of DNA extracted from blood collected during routine clinical testing and linked de-identified medical records.[6,7] Samples from more than 90,000 individuals were genotyped using the Illumina MEGA-Ex chip. Breast cancer cases were identified from the electronic medical record systems. In this study, we only kept adult participants of African ancestry.

Multiethnic Cohort (MEC): The MEC is a prospective cohort study conducted in Hawaii and the Los Angeles area and included 215,251 study participants recruited between 1993 and 1996.[8] African American study participants were recruited from the Los Angeles area. Data collection at

baseline included a detailed, self-administered questionnaire that obtained information on basic demographic variables and several life-style and medical variables that have been associated with cancer risk. Incident cancer cases were identified through two state-wide Surveillance, Epidemiology, and End Results (SEER) registries: the Hawaii Tumor Registry and the California State Cancer Registry. Blood samples were collected from a substantial portion of the cohort.

Women's Contraceptive and Reproductive Experiences Study (CARE): The Women's CARE Study is a large multi-center population-based case-control study sponsored by the National Institute of Child Health and Human Development (NICHD).[9] It was designed to examine the effects of oral contraceptive use on invasive breast cancer risk. Cases diagnosed with breast cancer between 34 and 64 years of age were recruited in five U.S. locations (Atlanta, Detroit, Los Angeles, Philadelphia, and Seattle). Cases in Los Angeles County were diagnosed from July 1, 1994, through April 30, 1998, and controls were sampled by random digit dialing from the same population and time period.

San Francisco Bay Area Cancer Study (SFBCS): The SFBCS is a population-based case control study of invasive breast cancer in Hispanic, African American, and non-Hispanic White females in the San Francisco Bay Area.[10] From 1995 to 2003, females aged 35-79 years, diagnosed with a first primary invasive breast cancer were identified through the California population-based Greater Bay Area Cancer Registry. Population controls were identified through random digit dialing.

Ghana Breast Health Study (GBHS): The GBHS is a population-based case-control study conducted in Accra and Kumasi, Ghana, between 2013 and 2015.[11,12] Breast cancer cases were identified from females recommended for biopsy of a breast lesion suspicious for malignancy at one of the three major cancer treatment hospitals in Ghana (Korle Bu Teaching Hospital in Accra, and Komfo Anokye Teaching Hospital, and Peace and Love Hospital in Kumasi); or females presenting for treatment of pathologically-confirmed breast cancer at Korle Bu, Komfo Anokye, or Peace and Love Hospitals within one year of diagnosis. Controls were frequency matched to cases by age and district of residence.

The United States Radiologic Technologists (USRT) cohort: The USRT is a cohort study for cancer incidence and mortality which recruited approximately 140,00 U.S. radiologic technologists who were certified for at least two years between 1926 and 1982.[13] Breast cancer cases were confirmed based on pathology or medical records.

The Prostate, Lung, Colorectal, and Ovarian Cancer Screening Trial (PLCO): The PLCO is a multicenter, two-armed, randomized trial designed to evaluate the screening efficacy for prostate, lung, colorectal and ovarian cancer.[14] It recruited approximately 155,000 men and females, aged 55-74 years, from 1993 to 2001.

Nigerian Breast Cancer Study (NBCS): The NBCS is an ongoing case-control study of breast cancer in Ibadan, Nigeria initiated in 1998.[15,16] Breast cancer cases were 20 years or older, ascertained at the University College Hospital, Ibadan, which is the oldest tertiary hospital in Nigerian with a catchment population of approximate three million. Controls were recruited from a randomly selected community in one of the communities adjoining the hospital. The majority of the study subjects were Yoruba and Yoruba is one of the populations selected by the International HapMap Project to represent African continent.

Chicago Cancer Prone Study (CCPS): The CCPS is a hospital-based case-control study designed to investigate the genetics of young-onset breast cancer. Cases with histologically confirmed breast cancer were enrolled through the Cancer Risk Clinic at the University of Chicago. Young-onset cases and African Americans were oversampled. Controls were gender- and age-matched with cases and enrolled from patients who visited the same hospital and were willing to donate blood for genetic studies.

Women of African Ancestry Breast Cancer Study (WAABCS): The WAABCS is a hospital-based case-control study originally started in Nigeria in 1998 and was expanded to Uganda and Cameroon in 2011 with the same questionnaires and protocol.[17,18]

Carolina Breast Cancer Study (CBCS): The CBCS is a population-based case-control study conducted in 24 counties of central and eastern North Carolina.[19] From 1993 to 2001, it recruited females aged between 20 and 74 years and diagnosed with invasive breast cancer. African American females and females aged less than 50 years were oversampled. Cases were identified

by rapid case ascertainment system in cooperation with the North Carolina Central Cancer Registry. Controls were selected from the North Carolina Division of Motor Vehicle (for females younger than 65 years) and United States Health Care Financing Administration (for females aged 65 and older). Controls were approximately frequency matched to cases by age and race. Blood samples were collected from participants with consent.

Women's Circle of Health Study (WCHS): The WCHS is a case-control study established in 2003 in the New York City metropolitan areas, and beginning in 2006, from 10 counties in New Jersey.[20] Eligible cases included females who were diagnosed with invasive breast cancer between 20 and 75 years of age and self-identified as European American or African American. Controls were initially identified through random digit dialing and were matched to cases by self-reported race and 5-year age categories. From 2009-2012, controls were recruited through community events, particularly through churches.[21]

Black Women's Health Study (BWHS): The BWHS is a prospective cohort study which recruited approximately 59,000 African American females, aged 21 - 60 years, from all regions of the United States in 1995.[22] Participants were enrolled by completing a postal health questionnaire and were followed by mail questionnaires every two years. DNA samples were obtained from BWHS participants (26,800 females) by the mouthwash-swish method with all samples stored in freezers at -80°C.

MD Anderson Breast Cancer Study (MDABCS): All breast cancer cases in MDABCS are newly registered, histologically confirmed breast cancer patients at MD Anderson Cancer Center.[23] Basic demographic and epidemiological information including smoking, alcohol, education, and family history data were collected as part of institutional patient history database. Clinical data were abstracted from electronic medical records by clinical coding specialists. DNA were extracted from residual blood samples and banked in the institutional Blood Specimen Research Resource.

Northern California Breast Cancer Family Registry (NC-BCFR): Incident breast cancer cases included females aged <65 years, identified through the SEER cancer registry of the Greater San Francisco Bay Area (diagnoses 1995-2009) and the Sacramento region (diagnoses

2005-2006).[24,25] All cases with indicators of inherited breast cancer were included. Among cases aged 35 - 64 years without such indicators, cases from racial and ethnic minority populations were oversampled. Controls were identified through random digit dialing and frequency matched to cases diagnosed from 1995 to 1998 on 5-year age group and race/ethnicity, at a ratio of one control per two cases.

The Sister Study (SISTER): The Sister Study is a prospective cohort study designed to address genetic and environmental risk factors for breast cancer by the National Institute of Environmental Health Sciences. From 2003 through 2009, 50,884 U.S. females, including and Puerto Ricans, were recruited through a national multimedia campaign and network of recruitment volunteers, breast cancer professionals, and advocates. Participants were females aged 35 to 74 years and had a sister diagnosed with breast cancer.[26] At enrollment, participants completed baseline questionnaires on medical and family history, lifestyle factors, and demographics. Blood samples were collected during a home visit by trained phlebotomists and shipped overnight to the Sister Study laboratory where they were processed to obtain serum and stored at -80°C.

The Two Sister Study (2SISTER): The Two Sister Study is a family-based retrospective study developed from the Sister Study. The Two Sister Study recruited the case sisters in the Sister Study who were diagnosed within 4 years and had been younger than age 50 years at diagnosis.[27]

Wake Forest University Breast Cancer Study (WFBC): The WFBC is a clinic-based case-control study at Wake Forest University Health Sciences from 1998 to 2008.[28,29] Incident breast cancer cases were recruited at the Wake Forest University Breast Care Center. Controls were recruited from the patient population receiving routine mammography at the Outpatient Radiology-Breast Screening Center. Blood samples (20 ml) were collected from all study subjects.

New York University Women's Health Study (NYUWHS): The NYUWHS is a cohort study which enrolled 14,274 females aged 34 to 65 years attending Guttman Breast Diagnostic Institute in New York City for yearly screening from 1985 to 1991.[30,31] Self-administered questionnaires were used to collect demographic, medical, anthropometric, reproductive, and dietary. Non-

fasting peripheral venous blood was drawn prior to breast examination and serum samples were stored at -80°C for subsequent biochemical analyses. Up until 1991, females who returned to the clinic for annual breast cancer screening were asked to donate blood at each of their visits. Cases were breast cancer patients arising from in the cohort, and controls were females selected from the same cohort who were not diagnosed with breast cancer and matched to cases on age and follow up time.

Barbados National Cancer Study (BNCS): The BNCS is a population-based case-control study of incident breast and prostate cancer in the predominantly African population of Barbados, West Indies.[32] Breast cancer cases were histologically confirmed incident cases identified through the only pathology department on the island, located at the Queen Elizabeth Hospital, between July 2002 and March 2006. Controls were selected from a national database provided by the Barbados Statistical Services Department, and were frequency matched to breast cancer cases at a 2:1 ratio and by 5-year age groups. Blood samples were collected from participants.

Racial Variability in Genotypic Determinants of Breast Cancer Risk Study (RVGBC): RVGBC is a hospital-based case-control study conducted in Philadelphia and Detroit metropolitan areas from 1999 to 2003. Breast cancer cases were identified in the University of Pennsylvania Health System and Karmanos Cancer Institute. Local advertisement was also put to recruit breast cancer cases living in the Philadelphia and Detroit area. Controls were recruited in the same way as cases except that they did not have breast cancer. Patients with breast cancer had to be diagnosed within 18 months of recruitment and have invasive ductal cancer. The study over-sampled females diagnosed with breast cancer under age of 40 years.

Baltimore Breast Cancer Study (BBCS): The BBCS is a case control study of breast cancer designed to identify and characterize markers of disease aggressiveness and poor outcome. From 1993 to 2003, incident breast cancer cases and controls were recruited from six hospitals in the greater Baltimore area, including the University of Maryland Medical Center, the Baltimore Veterans Affairs Medical Center, Union Memorial Hospital, Mercy Medical Center, and the Sinai Hospital. Controls were frequency matched to cases by race and age.

ACKNOWLEDGEMENTS (SUPPLEMENTARY MATERIALS)

Research supports for participating studies of the AABCG consortium are provided below:

The SCCS was supported by grant U01 CA202979 from the National Institute of Health (NIH). The NBHS was supported by NIH grant R01CA100374. The STSBHS was supported by NIH grants U54CA163069, U54CA163072 and R03CA192214 and the National Center for Advancing Translational Sciences (UL1TR000445). The BEST was supported by the National Cancer Institute (R01CA202981) and the Susan G. Komen Foundation (SAC210105). Vanderbilt University Medical Center's BioVU projects were supported by numerous sources: institutional funding, private agencies, and federal grants. These include NIH funded Shared Instrumentation Grant S10OD017985, S10RR025141, and S10OD025092; CTSA grants UL1TR002243, UL1TR000445, and UL1RR024975. Genomic data are also supported by investigator-led projects that include U01HG004798, R01NS032830, RC2GM092618, P50GM115305, U01HG006378, U19HL065962, R01HD074711. The BWHS was supported by NIH grants U01CA164974, NIH R01CA228357, Susan G. Komen SAC 220228, Karin Grunebaum Cancer Research Foundation. The MDABCS was supported in part from the Texas Tobacco Settlement Funds and the University of Texas MD Anderson Cancer Center Duncan Family Institute for Cancer Prevention and Risk Assessment. NBCS, WAABCS, and CCPS investigators were supported by National Institutes of Health (R01-CA89085, R01-CA142996, R01-CA228198, R01-CA242929, P20-CA233307, and R01-MD013452) and Breast Cancer Research Foundation (BCRF-22-071). The CBCS was supported by the University Cancer Research Fund of North Carolina, the Susan G Komen Foundation, the National Cancer Institute of the National Institutes of Health (P01CA151135), and the National Cancer Institute Specialized Program of Research Excellence (SPORE) in Breast Cancer (NIH/NCI P50-CA058223). The Northern California site of the Breast Cancer Family Registry (NC-BCFR) was funded by grant U01 CA164920 from the National Cancer Institute. The content of this manuscript does not necessarily reflect the views or policies of the National Cancer Institute or any of the collaborating centers in the Breast Cancer Family Registry (BCFR), nor does mention of trade names, commercial products, or organizations imply endorsement by the U.S. Government or the BCFR. The San Francisco Bay Area Breast Cancer Study (SFBCS) was supported by grants R01 CA63446 and R01 CA77305 from the National Cancer Institute, grant DAMD17-96-1-6071 from the U.S. Department of

Defense and grant 7PB-0068 from the California Breast Cancer Research Program. The Women's CARE Study was supported in part by grants from the Breast Cancer Research Foundation, Tower Cancer Research Foundation, a gift from Dr. Richard Balch, and an endowed chair, the Harold E. Lee Chair for Cancer Research, and the USC Norris Comprehensive Cancer Center (NCI Cancer Center Support Grant P30 CA014089). The SISTER study was supported by the Intramural Research Program of the National Institutes of Health, National Institute of Environmental Health Sciences (grants Z01-ES044005 and Z01-ES102245), Susan G. Komen for the Cure (grant FAS0703856). The BBCS was supported by NCI Center for Cancer Research Intramural Research Program (ZIA BC 010887). The NYUWHS was supported by NIH grant U01CA182934.

The GBHS project was funded with intramural funds from the National Cancer Institute, National Institutes of Health. GBHS would like to acknowledge the contribution from the following individuals to the GBHS: Korle Bu Teaching Hospital,Accra—Prof. Joe Nat Clegg-Lamptey, Dr. Florence Dedey, Dr. Lawrence Edusei, Dr. Verna Vanderpuye, Dr. Joel Yarney, Dr Adu-Aryee, Obed Ekpedzor, Angela Kenu, Victoria Okyne, Naomi Oyoe Ohene Oti, Evelyn Tay; Komfo Anoyke Teaching Hospital, Kumasi—Dr. Ernest Adjei, Dr. Francis Aitpillah, Dr. Daniel Ansong, Dr. Baffour Awuah, Dr. Joseph Oppong, Dr. Ernest Osei-Bonsu, Dr. Nicholas Titiloye, Marion Alcpaloo, Bernard Arhin, Emmanuel Asiamah, Isaac Boakye, Samuel Ka-chungu and; Peace and Love Hospital, Kumasi—Dr. Beatrice Addai Wiafe, Dr. Seth Wiafe, Samuel Amanama, Emma Abaidoo, Prince Agyapong, Thomas Agyei, Debora Boateng-Ansong, Margaret Frempong, Bridget Nortey Mensah, Richard Opoku, and Kofi Owusu Gyimah; University of Ghana, Accra—Prof. Richard Biritwum, Dr. Kofi Nyarko; and Dr. Jonine Figueroa of the University of Edinburg, Scotland. The study was further enhanced by the surgical expertise provided by Dr Lisa Newman of the University of Michigan and by pathological expertise provided by Drs. Stephen Hewitt and Petra Lenz of the National Cancer Institute and Dr. Maire A. Duggan from the Cumming School of Medicine, University of Calgary, Canada. Study management assistance was received from Ricardo Diaz, Shelley Niwa, Usha Singh, Ann Truelove and Michelle Brotzman at Westat, Inc. Appreciation is also expressed to the many females who agreed to participate in the study and to provide information and biospecimens in hopes of preventing and improving outcomes of breast cancer in Ghana. The GBHS also

acknowledges the research contributions of the Cancer Genomics Research Laboratory for their expertise, execution, and support of this research in the areas of project planning, wet laboratory processing of specimens, and bioinformatics analysis of generated data. This project has been funded in whole or in part with Federal funds from the National Cancer Institute, National Institutes of Health, under NCI Contract No. 75N910D00024. The content of this publication does not necessarily reflect the views or policies of the Department of Health and Human Services, nor does mention of trade names, commercial products, or organizations imply endorsement by the U.S. Government.

SUPPLEMENTARY REFERENCES

1. Signorello, L.B. *et al.* Southern community cohort study: establishing a cohort to investigate health disparities. *J Natl Med Assoc* **97**, 972-9 (2005).
2. Signorello, L.B., Hargreaves, M.K. & Blot, W.J. The Southern Community Cohort Study: investigating health disparities. *J Health Care Poor Underserved* **21**, 26-37 (2010).
3. Cui, Y. *et al.* Interactions of hormone replacement therapy, body weight, and bilateral oophorectomy in breast cancer risk. *Clin Cancer Res* **20**, 1169-78 (2014).
4. Sanderson, M. *et al.* A Pooled Case-only Analysis of Reproductive Risk Factors and Breast Cancer Subtype Among Black Women in the Southeastern United States. *Cancer Epidemiol Biomarkers Prev* **30**, 1416-1423 (2021).
5. Pal, T. *et al.* A high frequency of BRCA mutations in young black women with breast cancer residing in Florida. *Cancer* **121**, 4173-80 (2015).
6. Roden, D.M. *et al.* Development of a large-scale de-identified DNA biobank to enable personalized medicine. *Clin Pharmacol Ther* **84**, 362-9 (2008).
7. Kasimatis, K.R. *et al.* Evaluating human autosomal loci for sexually antagonistic viability selection in two large biobanks. *Genetics* **217**, 1-10 (2021).
8. Kolonel, L.N., Altshuler, D. & Henderson, B.E. The multiethnic cohort study: exploring genes, lifestyle and cancer risk. *Nat Rev Cancer* **4**, 519-27 (2004).
9. Marchbanks, P.A. *et al.* The NICHD Women's Contraceptive and Reproductive Experiences Study: methods and operational results. *Ann Epidemiol* **12**, 213-21 (2002).
10. John, E.M., Schwartz, G.G., Koo, J., Wang, W. & Ingles, S.A. Sun exposure, vitamin D receptor gene polymorphisms, and breast cancer risk in a multiethnic population. *Am J Epidemiol* **166**, 1409-19 (2007).
11. Brinton, L.A. *et al.* Design considerations for identifying breast cancer risk factors in a population-based study in Africa. *Int J Cancer* **140**, 2667-2677 (2017).
12. Nyante, S.J. *et al.* Recruiting population controls for case-control studies in sub-Saharan Africa: The Ghana Breast Health Study. *PLoS One* **14**, e0215347 (2019).
13. Bhatti, P. *et al.* Polymorphisms in DNA repair genes, ionizing radiation exposure and risk of breast cancer in U.S. Radiologic technologists. *Int J Cancer* **122**, 177-82 (2008).
14. Prorok, P.C. *et al.* Design of the Prostate, Lung, Colorectal and Ovarian (PLCO) Cancer Screening Trial. *Control Clin Trials* **21**, 273S-309S (2000).
15. Huo, D. *et al.* Parity and breastfeeding are protective against breast cancer in Nigerian women. *Br J Cancer* **98**, 992-6 (2008).
16. Huo, D. *et al.* Genetic polymorphisms in uridine diphospho-glucuronosyltransferase 1A1 and breast cancer risk in Africans. *Breast Cancer Res Treat* **110**, 367-76 (2008).
17. Adebamowo, C.A. *et al.* Obesity and height in urban Nigerian women with breast cancer. *Ann Epidemiol* **13**, 455-61 (2003).
18. Hou, N. *et al.* An epidemiologic investigation of physical activity and breast cancer risk in Africa. *Cancer Epidemiol Biomarkers Prev* **23**, 2748-56 (2014).
19. Newman, B. *et al.* The Carolina Breast Cancer Study: integrating population-based epidemiology and molecular biology. *Breast Cancer Res Treat* **35**, 51-60 (1995).
20. Ambrosone, C.B. *et al.* Conducting Molecular Epidemiological Research in the Age of HIPAA: A Multi-Institutional Case-Control Study of Breast Cancer in African-American and European-American Women. *J Oncol* **2009**, 871250 (2009).

21. Bandera, E.V. *et al.* Rethinking sources of representative controls for the conduct of case-control studies in minority populations. *BMC Med Res Methodol* **13**, 71 (2013).
22. Palmer, J.R. *et al.* Genetic susceptibility loci for subtypes of breast cancer in an African American population. *Cancer Epidemiol Biomarkers Prev* **22**, 127-34 (2013).
23. Wu, X. *et al.* Cohort Profile: The MD Anderson Cancer Patients and Survivors Cohort (MDA-CPSC). *Int J Epidemiol* **45**, 713-713f (2016).
24. John, E.M. *et al.* The Breast Cancer Family Registry: an infrastructure for cooperative multinational, interdisciplinary and translational studies of the genetic epidemiology of breast cancer. *Breast Cancer Res* **6**, R375-89 (2004).
25. John, E.M., Sangaramoorthy, M., Koo, J., Whittemore, A.S. & West, D.W. Enrollment and biospecimen collection in a multiethnic family cohort: the Northern California site of the Breast Cancer Family Registry. *Cancer Causes Control* **30**, 395-408 (2019).
26. Sandler, D.P. *et al.* The Sister Study Cohort: Baseline Methods and Participant Characteristics. *Environ Health Perspect* **125**, 127003 (2017).
27. Fei, C., Deroo, L.A., Sandler, D.P. & Weinberg, C.R. Fertility drugs and young-onset breast cancer: results from the Two Sister Study. *J Natl Cancer Inst* **104**, 1021-7 (2012).
28. Smith, T.R. *et al.* DNA-repair genetic polymorphisms and breast cancer risk. *Cancer Epidemiol Biomarkers Prev* **12**, 1200-4 (2003).
29. Smith, T.R. *et al.* Polygenic model of DNA repair genetic polymorphisms in human breast cancer risk. *Carcinogenesis* **29**, 2132-8 (2008).
30. Toniolo, P.G. *et al.* Endogenous hormones and breast cancer: a prospective cohort study. *Breast Cancer Res Treat* **18 Suppl 1**, S23-6 (1991).
31. Zeleniuch-Jacquotte, A. *et al.* Premenopausal serum androgens and breast cancer risk: a nested case-control study. *Breast Cancer Res* **14**, R32 (2012).
32. Nemesure, B. *et al.* Risk factors for breast cancer in a black population--the Barbados National Cancer Study. *Int J Cancer* **124**, 174-9 (2009).

SUPPLEMENTARY TABLES

Table S1. Participating studies of the African-ancestry Breast Cancer Genetic (AABCG) consortium.

Study Full Name	Study Acronym	Institution	PI Name	PI Email	Acknowledgement
Southern Community Cohort Study	SCCS	Vanderbilt University Medical Center	Wei Zheng	wei.zheng@vanderbilt.edu	The SCCS was supported by grant U01 CA202979 from the National Institute of Health
Nashville Breast Health Study	NBHS	Vanderbilt University Medical Center	Wei Zheng	wei.zheng@vanderbilt.edu	The NBHS was supported by NIH grant R01CA100374.
Southern Tri-State Breast Health Study	STSBHS	Meharry Medical College / Vanderbilt University Medical Center	Sanderson/Zheng	msanderson@mmc.edu	The STSBHS was supported by NIH grants U54CA163069, U54CA163072 and R03CA192214 and the National Center for Advancing Translational Sciences (UL1TR000445).
Black Women: Etiology and Survival of Triple-negative Breast Cancers Study	BEST	Vanderbilt University Medical Center	Tuya Pal	tuya.pal@vumc.org	The BEST was supported by the National Cancer Institute (R01CA202981) and the Susan G. Komen Foundation (SAC210105).
Vanderbilt Biobank (BioVU)	BioVU	Vanderbilt University Medical Center	Dan Roden/Jirong Long	Jirong.long@vumc.org	Vanderbilt University Medical Center's BioVU projects are supported by numerous sources: institutional funding, private agencies, and federal grants. These include NIH funded Shared Instrumentation Grant S10OD017985, S10RR025141, and S10OD025092; CTSA grants UL1TR002243, UL1TR000445, and UL1RR024975. Genomic data are also supported by investigator-led projects that include U01HG004798, R01NS032830, RC2GM092618, P50GM115305, U01HG006378, U19HL065962, R01HD074711.
Multiethnic Cohort	MEC	University of Southern California	Christopher Haiman	Christopher.Haiman@med.usc.edu	
Women's Contraceptive and Reproductive Experiences Study	CARE	University of Southern California	Mike Press	press@usc.edu	The Women's CARE Study was supported in part by grants from the Breast Cancer Research Foundation, Tower Cancer Research Foundation (Jessica M. Berman Senior Investigator Award), a gift from Dr. Richard Balch, and an endowed chair, the Harold E. Lee Chair for Cancer Research, and the USC Norris Comprehensive Cancer Center (NCI Cancer Center Support Grant P30 CA014089).
San Francisco Bay Area Cancer Study	SFBCS	Stanford University	Esther M. John	emjohn@stanford.edu	The San Francisco Bay Area Breast Cancer Study (SFBCS) was supported by grants R01 CA63446 (E.M. John) and R01 CA77305 (E.M. John) from the National Cancer Institute, grant DAMD17-96-1-6071 (E.M. John) from the U.S. Department of Defense and grant 7PB-0068 (E.M. John) from the California Breast Cancer Research Program.

Ghana Breast Health Study	GBHS	National Cancer Institute	Montserrat García-Closas	montserrat.garcia-closas@nih.gov	The GBHS project was funded with intramural funds from the National Cancer Institute, National Institutes of Health. GBHS would like to acknowledge the contribution from the following individuals to the GBHS: Korle Bu Teaching Hospital,Accra—Prof. Joe Nat Clegg-Lamptey, Dr. Florence Dedey, Dr. Lawrence Edusei, Dr. Verna Vanderpuye, Dr. Joel Yarney, Dr Adu-Aryee, Obed Ekpedzor, Angela Kenu, Victoria Okyne, Naomi Oyoe Ohene Oti, Evelyn Tay; Komfo Anoyke Teaching Hospital, Kumasi—Dr. Ernest Adjei, Dr. Francis Aitpillah, Dr. Daniel Ansong, Dr. Baffour Awuah, Dr. Joseph Oppong, Dr. Ernest Osei-Bonsu, Dr. Nicholas Titiloye, Marion Alcpaloo, Bernard Arhin, Emmanuel Asiamah, Isaac Boakye, Samuel Ka-chungu and; Peace and Love Hospital, Kumasi—Dr. Beatrice Addai Wiafe, Dr. Seth Wiafe, Samuel Amanama, Emma Abaidoo, Prince Agyapong, Thomas Agyei, Debora Boateng-Ansong, Margaret Frempong, Bridget Nortey Mensah, Richard Opoku, and Kofi Owusu Gyimah; University of Ghana, Accra—Prof. Richard Biritwum, Dr. Kofi Nyarko; and Dr. Jonine Figueroa of the University of Edinburg, Scotland. The study was further enhanced by the surgical expertise provided by Dr Lisa Newman of the University of Michigan and by pathological expertise provided by Drs. Stephen Hewitt and Petra Lenz of the National Cancer Institute and Dr. Maire A. Duggan from the Cumming School of Medicine, University of Calgary, Canada. Study management assistance was received from Ricardo Diaz, Shelley Niwa, Usha Singh, Ann Truelove and Michelle Brotzman at Westat, Inc. Appreciation is also expressed to the many females who agreed to participate in the study and to provide information and biospecimens in hopes of preventing and improving outcomes of breast cancer in Ghana. The GBHS also acknowledges the research contributions of the Cancer Genomics Research Laboratory for their expertise, execution, and support of this research in the areas of project planning, wet laboratory processing of specimens, and bioinformatics analysis of generated data. This project has been funded in whole or in part with Federal funds from the National Cancer Institute, National Institutes of Health, under NCI Contract No. 75N910D00024. The content of this publication does not necessarily reflect the views or policies of the Department of Health and Human Services, nor does mention of trade names, commercial products, or organizations imply endorsement by the U.S. Government.
US Radiologic Technologists Study	USRT	National Cancer Institute	Montserrat García-Closas	montserrat.garcia-closas@nih.gov	
Prostate, Lung, Colorectal, and Ovarian Cancer Screening Trial	PLCO	National Cancer Institute	Montserrat García-Closas	montserrat.garcia-closas@nih.gov	
Nigerian Breast Cancer Study	NBCS	The University of Chicago	Dezheng Huo	dhuo@health.bsd.uchicago.edu	

Chicago Cancer Prone Study	CCPS	The University of Chicago	Olufunmilayo I. Olopade	folopade@medicine.bsd.uchicago.edu	NBCS, WAABCS, and CCPS investigators were supported by National Institutes of Health (R01-CA89085, R01-CA142996, R01-CA228198, R01-CA242929, P20-CA233307, and R01-MD013452) and Breast Cancer Research Foundation (BCRF-22-071).
Women of African Ancestry Breast Cancer Study	WAABCS	The University of Chicago	Olufunmilayo I. Olopade	folopade@medicine.bsd.uchicago.edu	
Carolina Breast Cancer Study	CBCS	University of North Carolina at Chapel Hill	Melissa Troester	troester@unc.edu	The CBCS was supported by the University Cancer Research Fund of North Carolina, the Susan G Komen Foundation, the National Cancer Institute of the National Institutes of Health (P01CA151135), and the National Cancer Institute Specialized Program of Research Excellence (SPORE) in Breast Cancer (NIH/NCI P50-CA058223).
Women's Circle of Health Study	WCHS	Roswell Park Comprehensive Cancer Center	Song Yao	Song.Yao@RoswellPark.org	
Black Women's Health Study	BWHS	Boston University	Julie R Palmer	jpalmer@bu.edu	The BWHS was supported by NIH grants U01CA164974, NIH R01CA228357, Susan G. Komen SAC 220228 (JRP), Karin Grunebaum Cancer Research Foundation (JRP).
MD Anderson Breast Cancer Study	MDABCS	The University of Texas MD Anderson Cancer Center	Jian Gu	jiangu@mdanderson.org	The MDABCS was supported in part from the Texas Tobacco Settlement Funds and the University of Texas MD Anderson Cancer Center Duncan Family Institute for Cancer Prevention and Risk Assessment.
Northern California Breast Cancer Family Registry	NC-BCFR	Stanford University	Esther M. John	emjohn@stanford.edu	The Northern California site of the Breast Cancer Family Registry (NC-BCFR) was funded by grant U01 CA164920 from the National Cancer Institute. The content of this manuscript does not necessarily reflect the views or policies of the National Cancer Institute or any of the collaborating centers in the Breast Cancer Family Registry (BCFR), nor does mention of trade names, commercial products, or organizations imply endorsement by the U.S. Government or the BCFR.
The Sister Study	SISTER	National Institute of Environmental Health Sciences	Dale Sandler	sandler@niehs.nih.gov	The SISTER study was supported by the Intramural Research Program of the National Institutes of Health, National Institute of Environmental Health Sciences (grants Z01-ES044005 to D.P.S. and Z01-ES102245 to C.R.W.), Susan G. Komen for the Cure (grant FAS0703856 to C.R.W.).
The Two Sister Study	2SISTER	National Institute of Environmental Health Sciences	Dale Sandler	sandler@niehs.nih.gov	
Wake Forest University Breast Cancer Study	WFBC	The Wake Forest University	Jennifer Hu	jhu@med.miami.edu	
New York University Women’s Health Study	NYUWHS	NYU Grossman School of Medicine	Anne Zeleniuch-Jacquotte	Anne.Jacquotte@nyulangone.org	

Barbados National Cancer Study	BNCS	Stony Brook University	Barbara Nemesure	Barbara.Nemesure@stonybrookmedicine.edu	
Racial Variability in Genotypic Determinants of Breast Cancer Risk Study	RVGBC	University of Pennsylvania	Katherine Nathanson	knathans@upenn.edu	
Baltimore Breast Cancer Study	BBCS	National Cancer Institute	Stefan Ambs	ambss@mail.nih.gov	The BBCS was supported by NCI Center for Cancer Research Intramural Research Program (ZIA BC 010887). The NYUWHS was supported by NIH grant U01CA182934.

Table S2. Sample sizes of studies contributing to the genome-wide association analysis.

Dataset	Study	Case	Control	Case by subtype [a]		
				**ER Positive**	**ER Negative**	**TNBC**
**Whole genome sequencing data**						
WGS [b]	NBHS	91	16	29	62	32
	SCCS	321	376	172	147	77
	STSBHS	421	0	175	246	171
	GBHS	293	147	112	113	69
	MEC	211	119	126	73	5
WGS-2	SCCS	71	1,639	23	0	0
**Subtotal**		**1,408**	**2,297**	**637**	**641**	**354**
**MEGA genotyping data**						
Genotyped in Vanderbilt	NBHS [c]	138	147	60	0	0
	SCCS	708	678	281	104	50
	STSBHS [c]	692	683	565	77	36
	MDABCS [c]	1,294	1,222	700	309	220
	CCPS	366	279	242	103	69
	NBCS	695	376	56	162	82
	NC-BCFR [c]	185	213	106	53	35
	NYUWHS	72	58	33	11	5
Genotyped in Roswell Park	WCHS	1,326	851	891	368	235
	BWHS	1,282	1,879	752	334	191
Genotyped in USC	MEC	1,194	914	823	264	162
**Subtotal**		**7,952**	**7,300**	**4,509**	**1,785**	**1,085**
**Genotyping data from existing studies or consortia**						
AMBER [d]	BWHS	307	2,098	207	63	42
	CBCS	602	1	393	185	136
	WCHS	472	243	334	135	88
ROOT	SCCS	126	323	78	31	18
	NBCS	702	602	91	134	78
	CCPS	365	376	161	130	84
	RVGBC	143	254	27	25	0

	BBCS	94	102	44	44	0
	BNCS	92	227	0	0	0
	NBHS	255	161	130	35	20
	NC-BCFR	383	48	226	128	40
	CARE	254	204	126	84	31
	CBCS	614	570	263	308	193
AABC	MEC	578	888	332	144	45
	PLCO	54	112	14	6	2
	SFBCS	157	210	87	49	2
	WCHS	63	21	36	26	21
	WFBC	113	138	62	41	19
GBHS	GBHS	660	1,496	227	225	111
	NBHS	51	53	13	12	8
	CBCS	855	46	494	288	215
	NC-BCFR	69	0	34	25	16
	MEC	605	607	419	160	91
OncoArray [e]	PLCO	24	68	12	2	2
	2SISTER	42	0	27	14	12
	SISTER	130	163	77	24	18
	USRT	26	38	0	0	0
	WAABCS	308	292	19	65	47
BioVU	BioVU	115	2,522	0	0	0
BEST	BEST [f]	352	351	219	114	82
iCOGS	NBHS	19	42	6	1	0
	SCCS	44	251	0	0	0
**Subtotal**		**8,674**	**12,507**	**4,158**	**2,498**	**1,421**
**Total**		**18,034**	**22,104**	**9,304**	**4,924**	**2,860**

[a] Studies with subtype cases less than 10 were not included in subtype analyses.

[b] As a covariate in association analyses, study was adjusted as GBHS, MEC, and other studies in WGS dataset.

[c] Matched controls from SCCS.

[d] As a covariate in association analyses, study was adjusted as WCHS and other studies in AMBER dataset.

Table S3. Results from permutation tests and conditional analyses.

Breast Cancer Subtype[1]	Gene	Cytoband	Nearest Index SNP	$P_{Permutation}$	$Z_{Conditional}$	$P_{Conditional}$
**Exp-TWAS**						
Overall	CTD-3080P12.3	5p15.33	rs2853669	0.001	1.62	0.11
ER-Neg	EN1	2q14.2	rs76664032	9.3×10^{-28}	-5.03	5.0×10^{-7}
	LINC01956	2q14.2	rs76664032	3.9×10^{-25}	-3.41	6.4×10^{-4}
	CTD-3080P12.3	5p15.33	rs2853669	0.001	2.2	0.028
TNBC	EN1	2q14.2	rs76664032	1.5×10^{-28}	-5.07	4.0×10^{-7}
	LINC01956	2q14.2	rs76664032	5.1×10^{-24}	-3.38	7.1×10^{-4}
	MRPL34	19p13.1	rs4609972	0.001	0.23	0.82
**APA-WAS**						
Overall	TET2	4q24	rs62331150	5.1×10^{-10}	-1.35	0.18
ER-Neg	TET2	4q24	rs62331150	1.7×10^{-13}	-0.83	0.4
**spTWAS**						
Overall	BRD9	5p15.33	rs2853669	3.3×10^{-4}	-1.7	0.089
	NUP210L	1q21.3	/	9.7×10^{-13}	/	/
ER-Neg	BRD9	5p15.33	rs2853669	0.002	-1.85	0.065
TNBC	BRD9	5p15.33	rs2853669	1.0×10^{-4}	-1.14	0.25

P-values were derived from the Z score tests (two-sided). Statistical significant threshold for multiple comparison adjustment is defined as $P < 5.0 \times 10^{-6}$ for gene expression TWAS of 9,982 tests (*0.05/9,982*), $P < 1.5 \times 10^{-5}$ for APA-WAS of 3,309 tests (0.05/3,309), and $P < 4.3 \times 10^{-6}$ for spTWAS of 11,426 tests (0.05/11,426) using Bonferroni correction.

[1] ER-Pos: Estrogen receptor (ER)-positive; ER-Neg: ER-negative; TNBC: triple-negative breast cancer; TWAS: transcriptome wide association study; Exp-TWAS: gene expression TWAS; APA-WAS: alternative polyadenylation (APA)-wide association study; spTWAS: splicing TWAS.

Table S4. Association results for genes identified in our recent TWAS conducted among females of Asian and European ancestry.

ENSEMBL ID	Gene Symbol	Current Study				Previous Study (EUR/ASN)			
		Z Score	P Value	Prediction R^2	Subtype[a]	Model[b]	Z Score	P Value	Subtype[b]
ENSG00000243323.6	PTPRVP	-1.52	0.06	0.09	Overall	Exp-TWAS	6.67	2.52×10^{-11}	ER-Neg
ENSG00000227082.1	CH17-437K3.1	1.60	0.05	0.02	Overall	Exp-TWAS	-4.62	3.78×10^{-6}	Overall
ENSG00000267058.1	RP11-15A1.3	-0.88	0.19	0.02	Overall	Exp-TWAS	6.50	8.07×10^{-11}	Overall
ENSG00000198792.12	TMEM184B	-0.97	0.17	0.06	Overall	spTWAS	5.58	2.35×10^{-8}	Overall
ENSG00000266918.1	RP11-798G7.8	1.00	0.16	0.06	Overall	Exp-TWAS	-4.77	1.86×10^{-6}	Overall
ENSG00000175279.21	CENPS	0.47	0.32	0.06	Overall	Exp-TWAS	-7.03	2.03×10^{-12}	Overall
ENSG00000261603.1	PRSS46	0.63	0.26	0.25	Overall	Exp-TWAS	-4.63	3.68×10^{-6}	Overall
ENSG00000089639.10	GMIP	0.13	0.45	0.01	Overall	spTWAS	-4.74	2.15×10^{-6}	Overall
ENSG00000182362.13	YBEY	0.01	0.49	0.42	ER-Pos	Exp-TWAS	4.72	2.37×10^{-6}	Overall
ENSG00000232300.1	FAM215B	-0.03	0.49	0.05	Overall	Exp-TWAS	-4.79	1.64×10^{-6}	Overall
ENSG00000232987.1	LINC01219	-0.07	0.47	0.02	Overall	Exp-TWAS	-5.91	3.33×10^{-9}	Overall
ENSG00000136997.16	MYC	-0.11	0.46	0.02	ER-Neg	Exp-TWAS	-5.67	1.46×10^{-8}	Overall
ENSG00000184381.18	PLA2G6	-0.11	0.46	0.18	Overall	Exp-TWAS	-6.35	2.17×10^{-10}	Overall
ENSG00000239653.1	PSMD6-AS2	0.13	0.45	0.05	Overall	Exp-TWAS	5.43	5.70×10^{-8}	Overall
ENSG00000255284.1	AP006621.5	0.12	0.45	0.03	ER-Pos	Exp-TWAS	6.22	5.05×10^{-10}	Overall
ENSG00000120071.13	KANSL1	-0.17	0.43	0.09	Overall	spTWAS	-4.66	3.20×10^{-6}	Overall
ENSG00000248508.6	SRP14-AS1	-0.19	0.43	0.23	ER-Pos	Exp-TWAS	-4.80	1.55×10^{-6}	Overall
ENSG00000111581.9	NUP107	0.23	0.41	0.03	ER-Neg	spTWAS	5.04	4.59×10^{-7}	ER-Pos
ENSG00000176222.8	ZNF404	0.18	0.43	0.07	Overall	Exp-TWAS	6.41	1.48×10^{-10}	Overall
ENSG00000279672.1	CMB9-55F22.1	0.21	0.42	0.21	Overall	Exp-TWAS	6.65	2.99×10^{-11}	Overall
ENSG00000213366.12	GSTM2	-0.34	0.37	0.12	Overall	Exp-TWAS	-4.75	2.05×10^{-6}	Overall
ENSG00000173156.6	RHOD	0.34	0.37	0.52	ER-Pos	Exp-TWAS	4.78	1.74×10^{-6}	ER-Pos
ENSG00000185829.17	ARL17A	-0.32	0.37	0.18	ER-Neg	Exp-TWAS	-5.36	8.44×10^{-8}	Overall
ENSG00000214425.7	LRRC37A4P	0.32	0.38	0.25	Overall	Exp-TWAS	6.06	1.40×10^{-9}	Overall
ENSG00000130638.16	ATXN10	0.39	0.35	0.10	ER-Neg	Exp-TWAS	5.44	5.23×10^{-8}	Overall
ENSG00000266916.5	ZNF793-AS1	-0.45	0.33	0.01	Overall	Exp-TWAS	-4.94	7.64×10^{-7}	Overall
ENSG00000172500.12	FIBP	0.39	0.35	0.08	Overall	Exp-TWAS	5.63	1.78×10^{-8}	Overall
ENSG00000247934.4	RP11-967K21.1	0.41	0.34	0.02	Overall	Exp-TWAS	6.73	1.74×10^{-11}	Overall
ENSG00000279059.1	RP11-257O5.2	0.37	0.36	0.06	Overall	Exp-TWAS	7.79	6.86×10^{-15}	Overall
ENSG00000264070.1	DND1P1	-0.48	0.32	0.01	ER-Neg	Exp-TWAS	-6.20	5.56×10^{-10}	Overall
ENSG00000262539.1	RP11-259G18.3	-0.57	0.28	0.05	Overall	Exp-TWAS	-5.46	4.72×10^{-8}	Overall
ENSG00000203791.14	METTL10	-0.67	0.25	0.14	Overall	Exp-TWAS	-4.70	2.63×10^{-6}	Overall
ENSG00000224165.5	DNAJC27-AS1	0.63	0.26	0.06	Overall	Exp-TWAS	5.12	3.08×10^{-7}	Overall
ENSG00000065559.14	MAP2K4	0.66	0.26	0.02	Overall	spTWAS	4.99	6.06×10^{-7}	Overall

ENSG00000171943.11	SRGAP2C	-0.33	0.37	0.12	Overall	Exp-TWAS	-10.08	6.76×10^{-24}	Overall
ENSG00000262500.1	MAPK8IP1P1	-0.61	0.27	0.17	Overall	Exp-TWAS	-5.56	2.73×10^{-8}	Overall
ENSG00000117481.10	NSUN4	-0.75	0.23	0.05	Overall	spTWAS	-5.13	2.93×10^{-7}	ER-Pos
ENSG00000153094.22	BCL2L11	-0.83	0.20	0.03	ER-Pos	spTWAS	-4.76	1.94×10^{-6}	Overall
ENSG00000071967.11	CYBRD1	-0.83	0.20	0.14	Overall	Exp-TWAS	-4.77	1.88×10^{-6}	Overall
ENSG00000164068.15	RNF123	0.87	0.19	0.04	Overall	Exp-TWAS	4.63	3.62×10^{-6}	Overall
ENSG00000177595.17	PIDD1	0.57	0.29	0.11	Overall	Exp-TWAS	7.42	1.13×10^{-13}	Overall
ENSG00000226864.2	ATE1-AS1	0.96	0.17	0.08	Overall	Exp-TWAS	4.66	3.18×10^{-6}	ER-Pos
ENSG00000205464.11	ATP6AP1L	-0.74	0.23	0.50	ER-Pos	Exp-TWAS	-6.01	1.90×10^{-9}	Overall
ENSG00000175467.14	SART1	1.04	0.15	0.02	Overall	spTWAS	4.64	3.48×10^{-6}	Overall
ENSG00000163636.10	PSMD6	-1.06	0.15	0.01	Overall	spTWAS	-4.62	3.87×10^{-6}	Overall
ENSG00000160766.14	GBAP1	-0.75	0.23	0.21	Overall	Exp-TWAS	-6.56	5.49×10^{-11}	Overall
ENSG00000037474.14	NSUN2	-1.02	0.15	0.41	Overall	Exp-TWAS	-4.89	1.01×10^{-6}	Overall
ENSG00000263503.1	MAPK8IP1P2	-0.82	0.21	0.18	Overall	Exp-TWAS	-6.19	5.99×10^{-10}	Overall
ENSG00000263001.5	GTF2I	1.06	0.15	0.02	Overall	Exp-TWAS	4.87	1.09×10^{-6}	Overall
ENSG00000196547.14	MAN2A2	0.99	0.16	0.05	Overall	APA-WAS	5.26	1.47×10^{-7}	Overall
ENSG00000143622.10	RIT1	-1.11	0.13	0.02	Overall	Exp-TWAS	-5.79	7.23×10^{-9}	Overall
ENSG00000272858.1	CTA-292E10.8	-1.08	0.14	0.02	Overall	Exp-TWAS	-6.25	4.11×10^{-10}	Overall
ENSG00000105701.15	FKBP8	-1.13	0.13	0.01	Overall	spTWAS	-6.04	1.51×10^{-9}	Overall
ENSG00000259314.1	CTD-3065B20.3	-1.33	0.09	0.04	Overall	Exp-TWAS	-5.43	5.55×10^{-8}	Overall
ENSG00000204642.13	HLA-F	1.73	0.04	0.20	Overall	APA-WAS	4.75	2.05×10^{-6}	Overall
ENSG00000136824.18	SMC2	-1.49	0.07	0.02	Overall	spTWAS	-5.65	1.59×10^{-8}	Overall
ENSG00000158517.13	NCF1	1.75	0.04	0.07	ER-Pos	APA-WAS	4.90	9.37×10^{-7}	Overall
ENSG00000240875.5	LINC00886	-1.73	0.04	0.46	ER-Neg	Exp-TWAS	-4.99	5.94×10^{-7}	Overall
ENSG00000277053.4	GTF2IP1	-1.82	0.03	0.19	Overall	Exp-TWAS	-4.84	1.28×10^{-6}	Overall
ENSG00000108784.9	NAGLU	-1.75	0.04	0.01	Overall	spTWAS	-5.28	1.27×10^{-7}	Overall
ENSG00000134184.12	GSTM1	-1.96	0.03	0.10	Overall	spTWAS	-4.82	1.42×10^{-6}	Overall
ENSG00000225190.10	PLEKHM1	-1.65	0.05	0.01	ER-Neg	spTWAS	-5.88	4.13×10^{-9}	Overall
ENSG00000196275.13	GTF2IRD2	1.87	0.03	0.22	Overall	Exp-TWAS	5.22	1.83×10^{-7}	Overall
ENSG00000127990.15	SGCE	1.74	0.04	0.03	ER-Pos	APA-WAS	5.74	9.27×10^{-9}	Overall
ENSG00000238083.7	LRRC37A2	-1.78	0.04	0.12	ER-Neg	Exp-TWAS	-5.66	1.49×10^{-8}	Overall
ENSG00000169231.13	THBS3	1.96	0.02	0.08	ER-Pos	Exp-TWAS	5.29	1.26×10^{-7}	Overall
ENSG00000179023.8	KLHDC7A	-1.67	0.05	0.20	Overall	Exp-TWAS	-6.33	2.40×10^{-10}	Overall
ENSG00000196189.12	SEMA4A	1.73	0.04	0.05	TNBC	spTWAS	6.25	4.22×10^{-10}	Overall
ENSG00000188086.13	PRSS45	-2.03	0.02	0.12	ER-Neg	spTWAS	-5.41	6.29×10^{-8}	Overall
ENSG00000142655.12	PEX14	2.27	0.01	0.06	Overall	Exp-TWAS	4.86	1.15×10^{-6}	Overall
ENSG00000214078.12	CPNE1	-2.36	0.01	0.68	Overall	Exp-TWAS	-4.68	2.88×10^{-6}	Overall
ENSG00000127220.5	ABHD8	2.37	0.01	0.21	Overall	Exp-TWAS	4.75	2.07×10^{-6}	Overall
ENSG00000255464.1	SEPT14P8	-1.91	0.03	0.01	ER-Pos	Exp-TWAS	-6.00	1.98×10^{-9}	ER-Neg
ENSG00000130511.15	SSBP4	1.01	0.16	0.04	Overall	spTWAS	11.49	1.47×10^{-30}	Overall

ENSG00000110917.7	MLEC	2.14	0.02	0.02	ER-Neg	spTWAS	5.57	2.61×10^{-8}	Overall
ENSG00000174912.7	METTL15P1	-2.37	0.01	0.06	ER-Neg	Exp-TWAS	-5.13	2.83×10^{-7}	Overall
ENSG00000173727.12	CMB9-22P13.1	2.19	0.01	0.06	ER-Neg	Exp-TWAS	5.64	1.74×10^{-8}	Overall
ENSG00000152348.15	ATG10	-1.93	0.03	0.03	ER-Pos	APA-WAS	-6.67	2.50×10^{-11}	Overall
ENSG00000186468.12	RPS23	2.14	0.02	0.50	Overall	Exp-TWAS	6.06	1.38×10^{-9}	Overall
ENSG00000155749.12	ALS2CR12	1.89	0.03	0.02	Overall	spTWAS	8.37	5.53×10^{-17}	Overall
ENSG00000175489.9	LRRC25	1.91	0.03	0.02	Overall	Exp-TWAS	9.47	2.84×10^{-21}	Overall
ENSG00000166263.13	STXBP4	1.87	0.03	0.03	Overall	spTWAS	9.97	2.15×10^{-23}	Overall
ENSG00000271828.1	CTD-2310F14.1	1.28	0.10	0.11	Overall	Exp-TWAS	14.92	2.35×10^{-50}	Overall
ENSG00000140400.16	MAN2C1	-3.31	4.7×10^{-4}	0.52	Overall	Exp-TWAS	-5.94	2.83×10^{-9}	Overall
ENSG00000064012.21	CASP8	-2.42	0.01	0.26	Overall	Exp-TWAS	-8.49	2.02×10^{-17}	Overall
ENSG00000166965.12	RCCD1	-2.60	0.005	0.08	Overall	Exp-TWAS	-10.87	1.60×10^{-27}	Overall

P-values were derived from the Z score tests (one-sided).

[a] ER-Pos: ER-positive; ER-Neg: ER-negative; TNBC: triple-negative breast cancer;

[b] TWAS: transcriptome wide association study; Exp-TWAS: gene expression TWAS; APA-WAS: alternative polyadenylation (APA)-wide association study; spTWAS: splicing TWAS.

Table S5. Association results for the lead variants within ±500Kb regions of the 29 previously reported breast cancer-associated genes replicated in this study, results from the African-ancestry Breast Cancer Genetic Study.

Gene	Breast Cancer Subtype	Lead Variant	Cytoband	POS (hg38)	Allele[a]	EAF[b]	OR (95% CI)	P Value
ABHD8	Overall	rs56069439	19p13.11	17283116	A/C	0.23	1.13 (1.09, 1.17)	1.10×10^{-11}
ALS2CR12	Overall	rs7588576	2q33.1	201251465	T/C	0.78	1.07 (1.03, 1.11)	4.70×10^{-4}
ATG10	ER Negative	rs224939	5q14.1	82421283	A/C	0.10	1.1 (1.04, 1.16)	1.03×10^{-3}
CASP8	Overall	rs7588576	2q33.1	201251465	T/C	0.78	1.07 (1.03, 1.11)	4.70×10^{-4}
CMB9-22P13.1	ER Negative	rs115650983	11q13.1	65553135	A/G	0.99	0.7 (0.54, 0.89)	4.23×10^{-3}
CPNE1	Overall	rs146367992	20q11.22	35701728	A/T	0.14	1.08 (1.03, 1.12)	1.05×10^{-3}
GSTM1	Overall	rs17024629	1p13.3	109637134	T/C	0.14	0.92 (0.87, 0.96)	1.50×10^{-4}
GTF2IP1	Overall	rs58869666	7q11.23	75724922	A/G	0.07	0.91 (0.86, 0.97)	2.24×10^{-3}
GTF2IRD2	Overall	rs76571753	7q11.23	74560585	T/G	0.06	0.91 (0.85, 0.97)	5.34×10^{-3}
HLA-F	Overall	rs9257930	6p22.1	29650917	A/C	0.02	1.26 (1.11, 1.43)	3.52×10^{-4}
KLHDC7A	Overall	rs79568179	1p36.13	18480914	C/G	0.95	0.88 (0.82, 0.94)	3.18×10^{-4}
LINC00886	ER Negative	rs9809108	3q25.31	156852167	A/T	0.81	1.12 (1.05, 1.19)	4.56×10^{-4}
LRRC25	Overall	rs184466283	19p13.11	18531491	A/G	0.04	0.85 (0.78, 0.93)	2.47×10^{-4}
LRRC37A2	ER Negative	rs197932	17q21.31	46896981	T/C	0.17	0.89 (0.83, 0.96)	2.57×10^{-3}
MAN2C1	Overall	rs4486847	15q24.2	75436190	C/G	0.45	1.07 (1.04, 1.1)	2.19×10^{-5}
METTL15P1	ER Negative	rs9809108	3q25.31	156852167	A/T	0.81	1.12 (1.05, 1.19)	4.56×10^{-4}
MLEC	ER Negative	rs150131933	12q24.31	120399345	A/G	0.98	0.72 (0.61, 0.87)	4.25×10^{-4}
NAGLU	Overall	rs116734341	17q21.2	42258814	T/C	0.96	1.15 (1.06, 1.24)	4.02×10^{-4}
NCF1	ER Positive	rs9638186	7q11.23	74369283	A/G	0.14	0.91 (0.85, 0.96)	7.64×10^{-4}
PEX14	Overall	rs74052157	1p36.22	10623109	A/G	0.07	1.14 (1.07, 1.2)	1.02×10^{-5}
PLEKHM1	ER Negative	rs81189	17q21.31	45817432	C/G	0.34	1.1 (1.05, 1.16)	2.58×10^{-4}
PRSS45	ER Negative	rs112296371	3p21.31	47194994	T/G	0.99	0.71 (0.59, 0.87)	9.21×10^{-4}
RCCD1	Overall	rs12594752	15q26.1	90988765	T/C	0.15	0.89 (0.85, 0.93)	5.35×10^{-7}
RPS23	Overall	rs224939	5q14.2	82421283	A/C	0.10	1.1 (1.04, 1.16)	1.03×10^{-3}
SEMA4A	TNBC	rs150077562	1q22	156497358	C/G	0.01	1.54 (1.19, 1.99)	9.09×10^{-4}
SEPT14P8	ER Positive	rs114005238	8p23.3	697791	A/T	0.04	0.83 (0.74, 0.92)	7.58×10^{-4}
SGCE	ER Positive	rs149667535	7q21.3	94801829	A/G	0.92	0.85 (0.79, 0.91)	2.41×10^{-6}
STXBP4	Overall	rs74773504	17q22	54956754	A/G	0.02	1.18 (1.07, 1.3)	6.33×10^{-4}
THBS3	ER Positive	rs138509259	1q22	154903810	C/G	0.98	1.33 (1.14, 1.55)	3.49×10^{-4}

[a] Effect allele/other allele;

[b] Effect allele frequency among controls.

Table S6. Genetic ancestry estimation of all the samples from the Susan G. Komen Normal Tissue Bank used for the model building.

Sample ID	AFR Prop	EAS Prop	EUR Prop	Sample ID	AFR Prop	EAS Prop	EUR Prop
VUMCKM_0668	0.9	0.02	0.09	VUMCKM_2506	0.92	0.01	0.07
VUMCKM_0771	0.85	0.03	0.12	VUMCKM_2511	0.83	0.01	0.17
VUMCKM_0950	0.87	0	0.13	VUMCKM_2517	0.85	0.01	0.14
VUMCKM_0964	0.82	0.01	0.17	VUMCKM_2518	0.82	0.02	0.16
VUMCKM_0973	0.89	0	0.11	VUMCKM_2519	0.92	0.02	0.07
VUMCKM_0976	0.91	0.01	0.08	VUMCKM_2531	0.85	0	0.15
VUMCKM_0982	0.85	0.04	0.11	VUMCKM_2532	0.84	0.01	0.15
VUMCKM_0986	0.91	0.01	0.08	VUMCKM_2535	0.87	0.03	0.1
VUMCKM_0987	0.81	0.03	0.16	VUMCKM_2536	0.91	0.03	0.06
VUMCKM_0989	0.84	0.01	0.15	VUMCKM_2547	0.86	0	0.14
VUMCKM_0991	0.88	0.01	0.1	VUMCKM_2564	0.89	0.03	0.08
VUMCKM_0997	0.89	0.02	0.09	VUMCKM_2568	0.82	0.04	0.14
VUMCKM_1016	0.86	0	0.14	VUMCKM_2571	0.82	0.03	0.15
VUMCKM_1021	0.91	0.02	0.07	VUMCKM_2595	0.86	0.02	0.12
VUMCKM_1024	0.88	0.01	0.11	VUMCKM_2603	0.86	0	0.14
VUMCKM_1042	0.83	0.03	0.14	VUMCKM_2606	0.82	0.01	0.17
VUMCKM_1046	0.82	0.01	0.17	VUMCKM_2608	0.88	0	0.11
VUMCKM_1070	0.88	0.01	0.11	VUMCKM_2614	0.89	0.03	0.08
VUMCKM_1072	0.86	0.01	0.13	VUMCKM_2615	0.83	0.01	0.16
VUMCKM_1091	0.82	0.01	0.17	VUMCKM_2675	0.87	0.03	0.11
VUMCKM_1099	0.89	0.05	0.06	VUMCKM_2678	0.82	0	0.18
VUMCKM_1111	0.91	0.02	0.07	VUMCKM_2679	0.89	0.02	0.09
VUMCKM_1120	0.9	0	0.09	VUMCKM_2712	0.92	0.01	0.06
VUMCKM_1155	0.82	0.02	0.15	VUMCKM_2718	0.88	0.01	0.1
VUMCKM_1169	0.84	0.04	0.12	VUMCKM_2720	0.87	0	0.13
VUMCKM_1174	0.85	0.02	0.13	VUMCKM_2786	0.92	0.02	0.05
VUMCKM_1175	0.84	0.02	0.13	VUMCKM_2820	0.87	0.03	0.1
VUMCKM_1183	0.91	0.02	0.08	VUMCKM_2845	0.82	0.01	0.16
VUMCKM_1236	0.83	0	0.17	VUMCKM_2846	0.86	0.04	0.1
VUMCKM_1256	0.84	0.01	0.15	VUMCKM_2895	0.83	0.03	0.14
VUMCKM_1297	0.84	0.01	0.16	VUMCKM_2908	0.87	0.01	0.12
VUMCKM_1298	0.91	0.02	0.07	VUMCKM_2912	0.85	0	0.15
VUMCKM_1311	0.9	0.01	0.09	VUMCKM_2920	0.88	0	0.12
VUMCKM_1312	0.87	0	0.13	VUMCKM_2938	0.88	0.03	0.09
VUMCKM_1351	0.84	0.02	0.14	VUMCKM_2942	0.93	0.03	0.04
VUMCKM_1361	0.84	0.03	0.13	VUMCKM_2943	0.83	0.01	0.16

VUMCKM_1372	0.89	0.01	0.1	VUMCKM_2950	0.89	0.01	0.09
VUMCKM_1375	0.84	0.01	0.15	VUMCKM_2956	0.9	0	0.09
VUMCKM_1376	0.81	0	0.19	VUMCKM_2957	0.86	0.01	0.13
VUMCKM_1385	0.83	0.01	0.16	VUMCKM_3026	0.81	0.01	0.17
VUMCKM_1464	0.91	0.02	0.07	VUMCKM_3057	0.84	0.03	0.13
VUMCKM_1471	0.9	0.01	0.09	VUMCKM_3070	0.8	0	0.2
VUMCKM_1549	0.86	0.03	0.12	VUMCKM_3090	0.84	0.02	0.14
VUMCKM_1558	0.88	0.02	0.09	VUMCKM_3108	0.9	0	0.1
VUMCKM_1560	0.9	0.01	0.09	VUMCKM_3127	0.88	0.04	0.08
VUMCKM_1589	0.86	0.02	0.11	VUMCKM_3135	0.87	0.01	0.12
VUMCKM_1611	0.88	0.03	0.09	VUMCKM_3162	0.88	0.01	0.11
VUMCKM_1615	0.92	0.02	0.06	VUMCKM_3164	0.87	0.02	0.11
VUMCKM_1619	0.81	0.02	0.17	VUMCKM_3203	0.83	0.02	0.15
VUMCKM_1627	0.9	0.03	0.07	VUMCKM_3231	0.89	0	0.11
VUMCKM_1636	0.93	0.01	0.06	VUMCKM_3240	0.83	0.02	0.16
VUMCKM_1638	0.86	0.02	0.12	VUMCKM_3247	0.87	0.02	0.11
VUMCKM_1653	0.88	0.03	0.09	VUMCKM_3269	0.83	0.01	0.16
VUMCKM_1655	0.82	0.04	0.14	VUMCKM_3277	0.9	0.02	0.08
VUMCKM_1751	0.86	0.03	0.11	VUMCKM_3365	0.84	0.02	0.13
VUMCKM_1800	0.86	0.02	0.12	VUMCKM_3379	0.83	0.02	0.15
VUMCKM_1806	0.87	0.01	0.12	VUMCKM_3417	0.91	0.01	0.08
VUMCKM_1807	0.86	0.02	0.12	VUMCKM_3436	0.87	0.02	0.11
VUMCKM_1810	0.88	0.01	0.1	VUMCKM_3439	0.82	0.02	0.16
VUMCKM_1853	0.89	0.01	0.1	VUMCKM_3464	0.9	0.03	0.07
VUMCKM_1913	0.86	0.02	0.13	VUMCKM_3506	0.85	0.02	0.13
VUMCKM_1929	0.88	0	0.12	VUMCKM_3518	0.88	0.04	0.09
VUMCKM_1933	0.9	0.03	0.07	VUMCKM_3524	0.88	0.01	0.1
VUMCKM_1934	0.88	0.02	0.1	VUMCKM_3527	0.81	0.02	0.18
VUMCKM_1937	0.87	0.01	0.12	VUMCKM_3530	0.9	0.03	0.07
VUMCKM_1939	0.89	0.02	0.1	VUMCKM_3535	0.93	0	0.07
VUMCKM_1979	0.9	0.03	0.07	VUMCKM_3540	0.86	0	0.14
VUMCKM_1980	0.9	0.02	0.08	VUMCKM_3546	0.85	0.02	0.13
VUMCKM_1994	0.87	0	0.13	VUMCKM_3568	0.91	0	0.09
VUMCKM_2193	0.89	0.02	0.09	VUMCKM_3590	0.82	0	0.17
VUMCKM_2203	0.89	0.02	0.08	VUMCKM_3610	0.99	0.01	0
VUMCKM_2262	0.84	0.01	0.15	VUMCKM_3614	0.82	0.03	0.15
VUMCKM_2264	0.9	0.03	0.07	VUMCKM_3638	0.81	0.02	0.17
VUMCKM_2462	0.82	0.03	0.16	VUMCKM_3640	0.86	0.01	0.12
VUMCKM_2503	0.89	0.02	0.09	VUMCKM_3653	0.87	0.04	0.09

SUPPLEMENTARY FIGURES

Figure S1. eQTpLot for gene CTD-3080P12.3 with overall breast cancer risk from Exp-TWAS. eQTpLot plots show the colocalization between eQTLs for gene CTD-3080P12.3 and GWAS signals for overall breast cancer risk. **A**: Locus with CTD-3080P12.3 gene. Points show variant p-values for overall breast cancer risk (vertical axis) and CTD-3080P12.3 expression (color scale). Triangles indicate GWAS effect direction and eQTL effect size. The genome-wide significance threshold (5×10^{-8}) is shown as a red line. **B:** Genomic positions of all genes within the locus. **C:** Enrichment of CTD-3080P12.3 eQTLs among GWAS-significant variants. **D:** Correlation between P_{GWAS} and P_{eQTL} for CTD-3080P12.3 and overall breast cancer risk, with the computed Pearson correlation coefficient (r) and p-value (p) displayed on the plot.

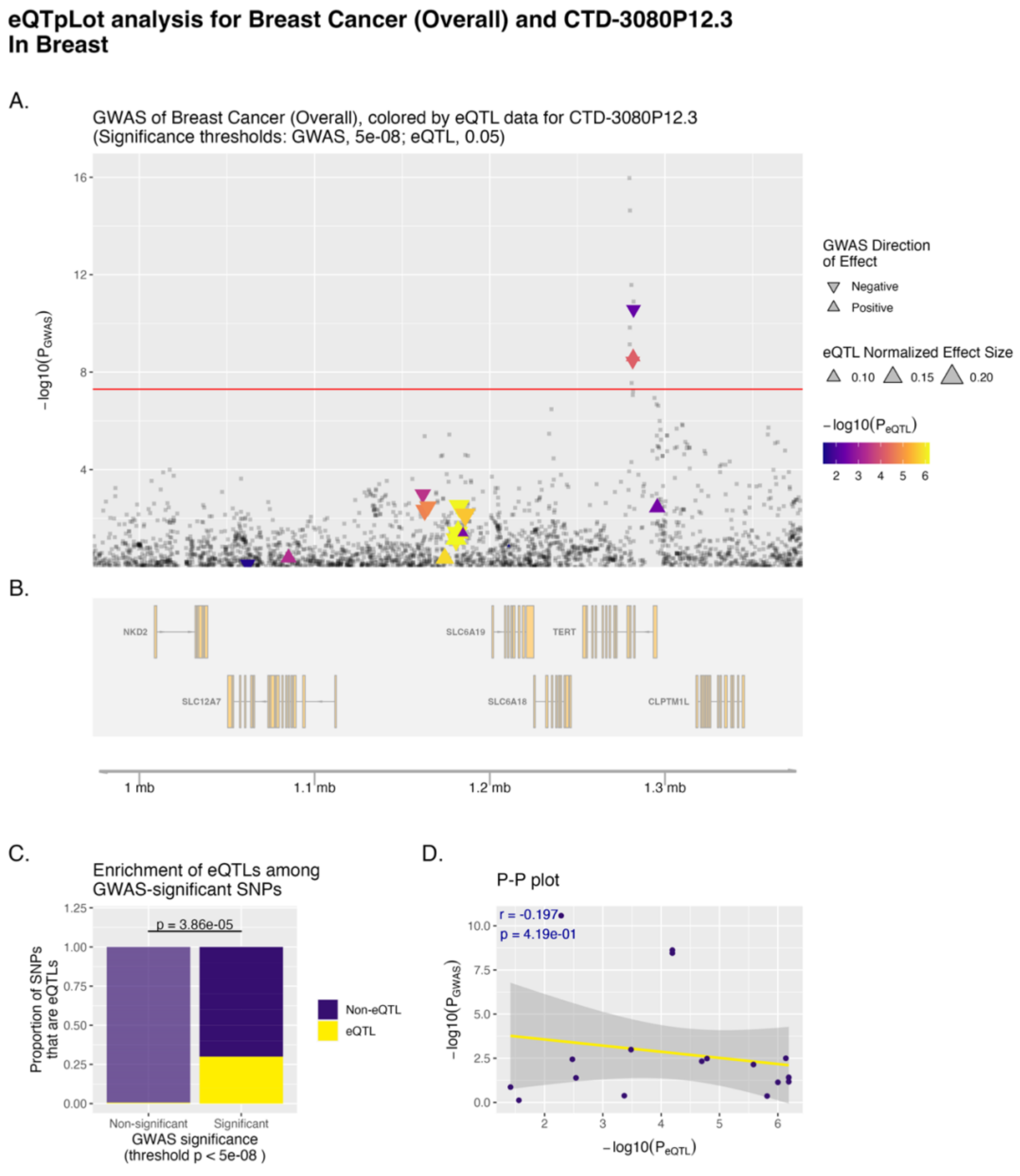

Figure S2. eQTpLot for gene EN1 with ER-negative breast cancer risk from Exp-TWAS. eQTpLot plots show the colocalization between eQTLs for gene EN1 and GWAS signals for ER-negative breast cancer risk. **A**: Locus with EN1 gene. Points show variant p-values for ER-negative breast cancer risk (vertical axis) and EN1 expression (color scale). Triangles indicate GWAS effect direction and eQTL effect size. The genome-wide significance threshold (5×10^{-8}) is shown as a red line. **B:** Genomic positions of all genes within the locus. **C:** Enrichment of EN1 eQTLs among GWAS-significant variants. **D:** Correlation between P_{GWAS} and P_{eQTL} for EN1 and ER-negative breast cancer risk, with the computed Pearson correlation coefficient (r) and p-value (p) displayed on the plot.

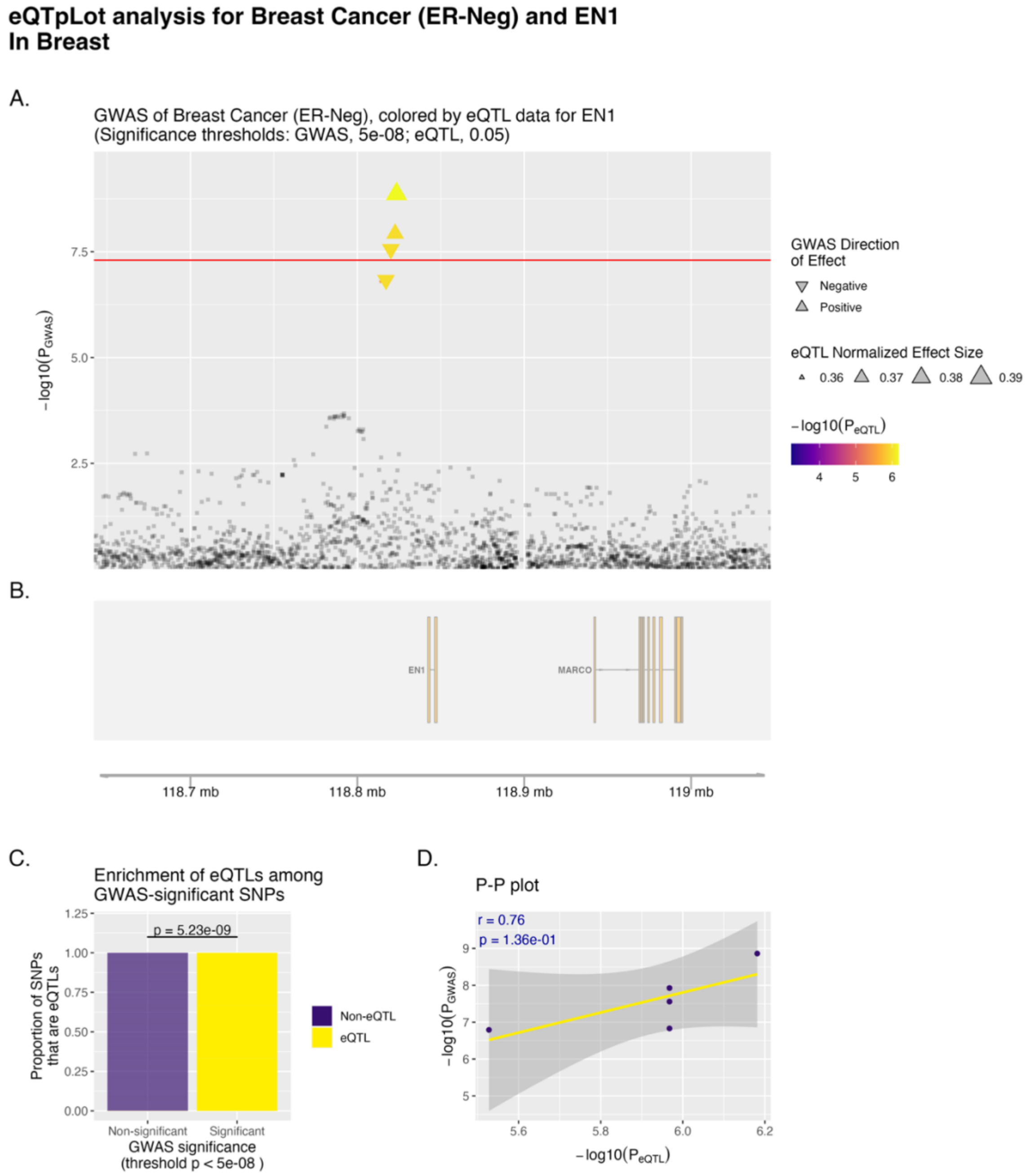

Figure S3. eQTpLot for gene LINC01956 with ER-negative breast cancer risk from Exp-TWAS. eQTpLot plots show the colocalization between eQTLs for gene LINC01956 and GWAS signals for ER-negative breast cancer risk. **A**: Locus with LINC01956 gene. Points show variant p-values for ER-negative breast cancer risk (vertical axis) and LINC01956 expression (color scale). Triangles indicate GWAS effect direction and eQTL effect size. The genome-wide significance threshold (5×10^{-8}) is shown as a red line. **B:** Genomic positions of all genes within the locus. **C:** Enrichment of LINC01956 eQTLs among GWAS-significant variants. **D:** Correlation between P_{GWAS} and P_{eQTL} for LINC01956 and ER-negative breast cancer risk, with the computed Pearson correlation coefficient (r) and p-value (p) displayed on the plot.

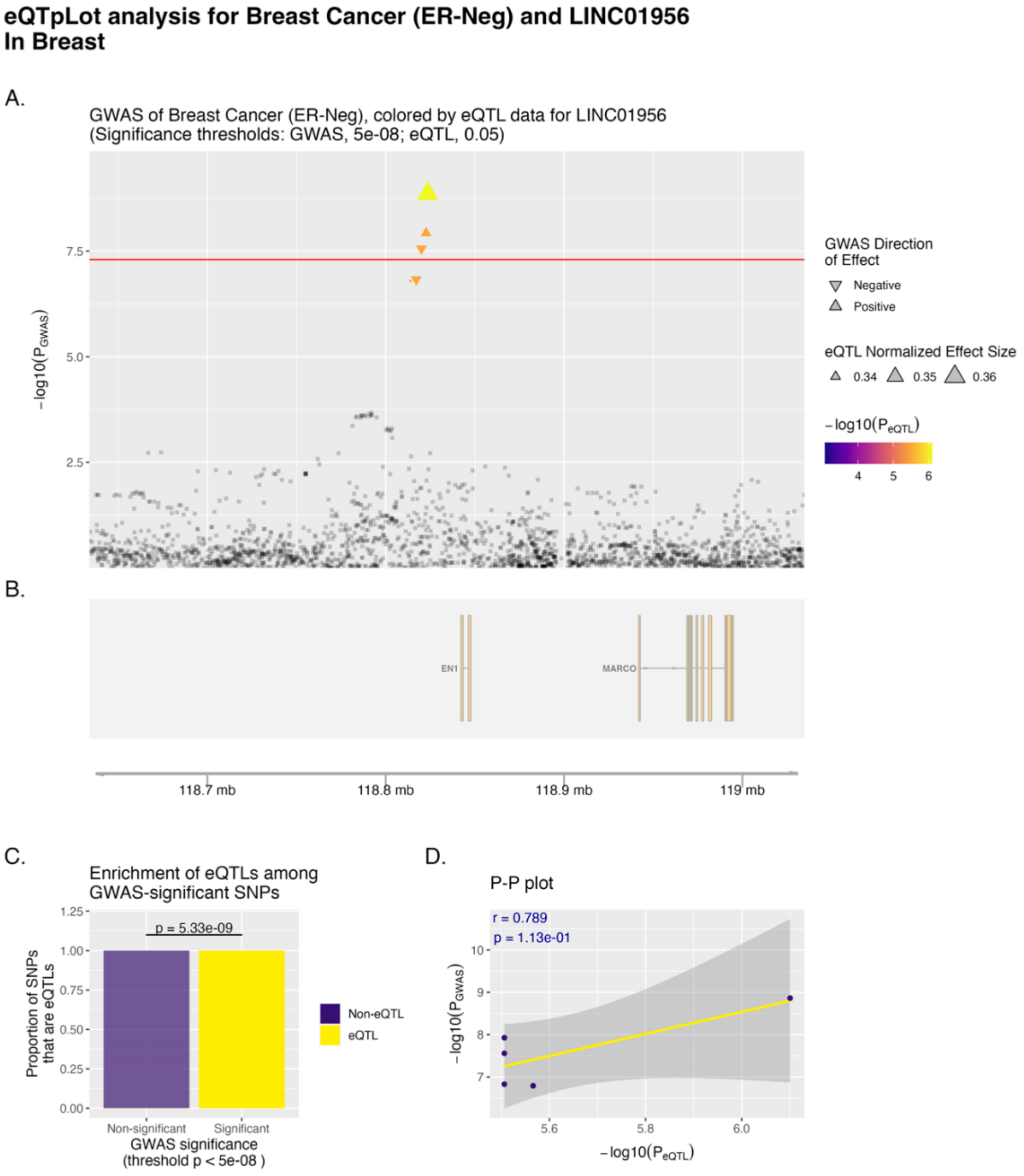

Figure S4. eQTpLot for gene CTD-3080P12.3 with ER-negative breast cancer risk from Exp-TWAS. eQTpLot plots show the colocalization between eQTLs for gene CTD-3080P12.3 and GWAS signals for ER-negative breast cancer risk. **A**: Locus with CTD-3080P12.3 gene. Points show variant p-values for ER-negative breast cancer risk (vertical axis) and CTD-3080P12.3 expression (color scale). Triangles indicate GWAS effect direction and eQTL effect size. The genome-wide significance threshold ($5{\times}10^{-8}$) is shown as a red line. **B:** Genomic positions of all genes within the locus. **C:** Enrichment of CTD-3080P12.3 eQTLs among GWAS-significant variants. **D:** Correlation between P_{GWAS} and P_{eQTL} for CTD-3080P12.3 and ER-negative breast cancer risk, with the computed Pearson correlation coefficient (r) and p-value (p) displayed on the plot.

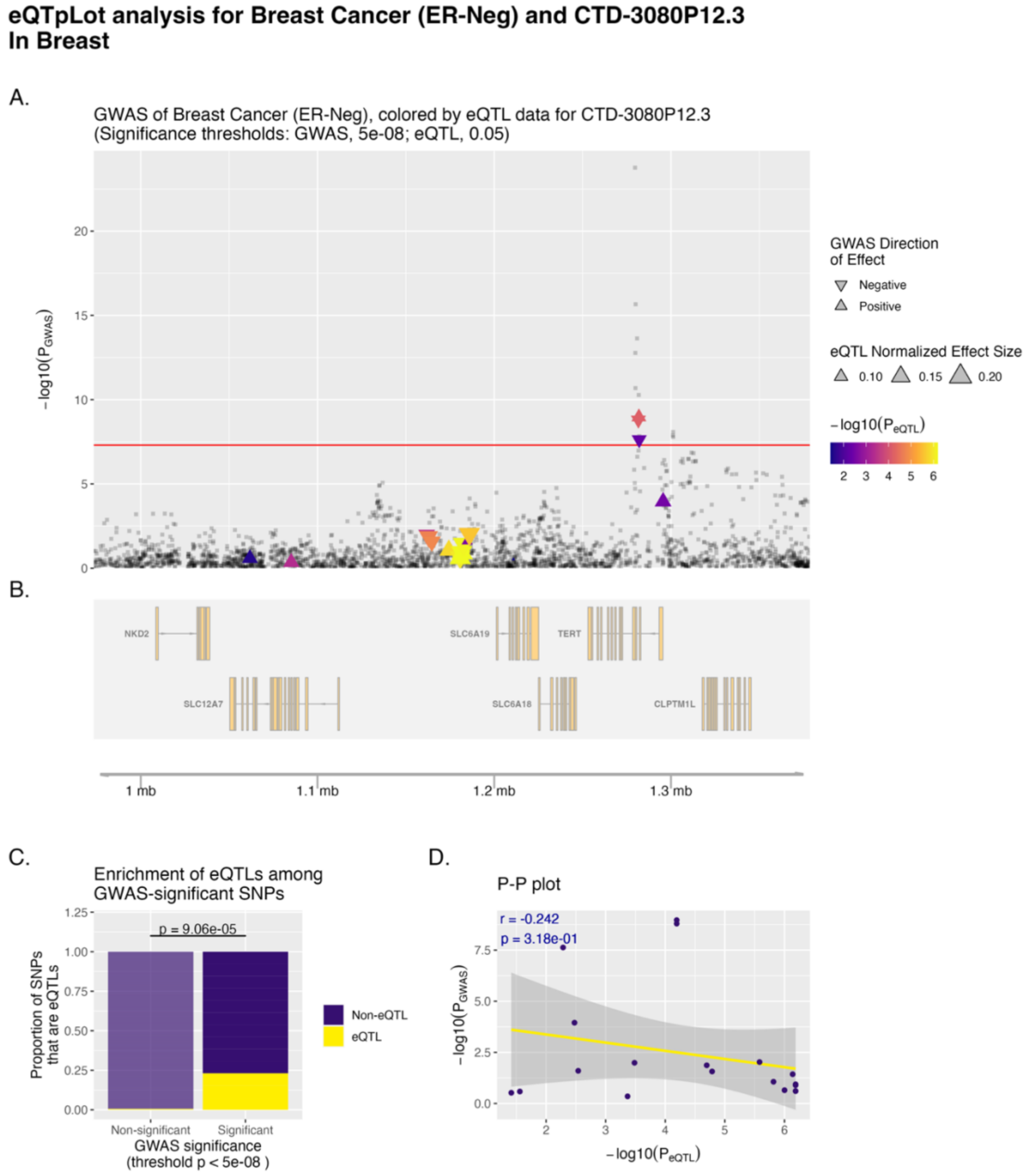

Figure S5. eQTpLot for gene EN1 with TNBC breast cancer risk from Exp-TWAS. eQTpLot plots show the colocalization between eQTLs for gene EN1 and GWAS signals for TNBC breast cancer risk. **A**: Locus with EN1 gene. Points show variant p-values for TNBC breast cancer risk (vertical axis) and EN1 expression (color scale). Triangles indicate GWAS effect direction and eQTL effect size. The genome-wide significance threshold (5×10^{-8}) is shown as a red line. **B:** Genomic positions of all genes within the locus. **C:** Enrichment of EN1 eQTLs among GWAS-significant variants. **D:** Correlation between P_{GWAS} and P_{eQTL} for EN1 and TNBC breast cancer risk, with the computed Pearson correlation coefficient (r) and p-value (p) displayed on the plot.

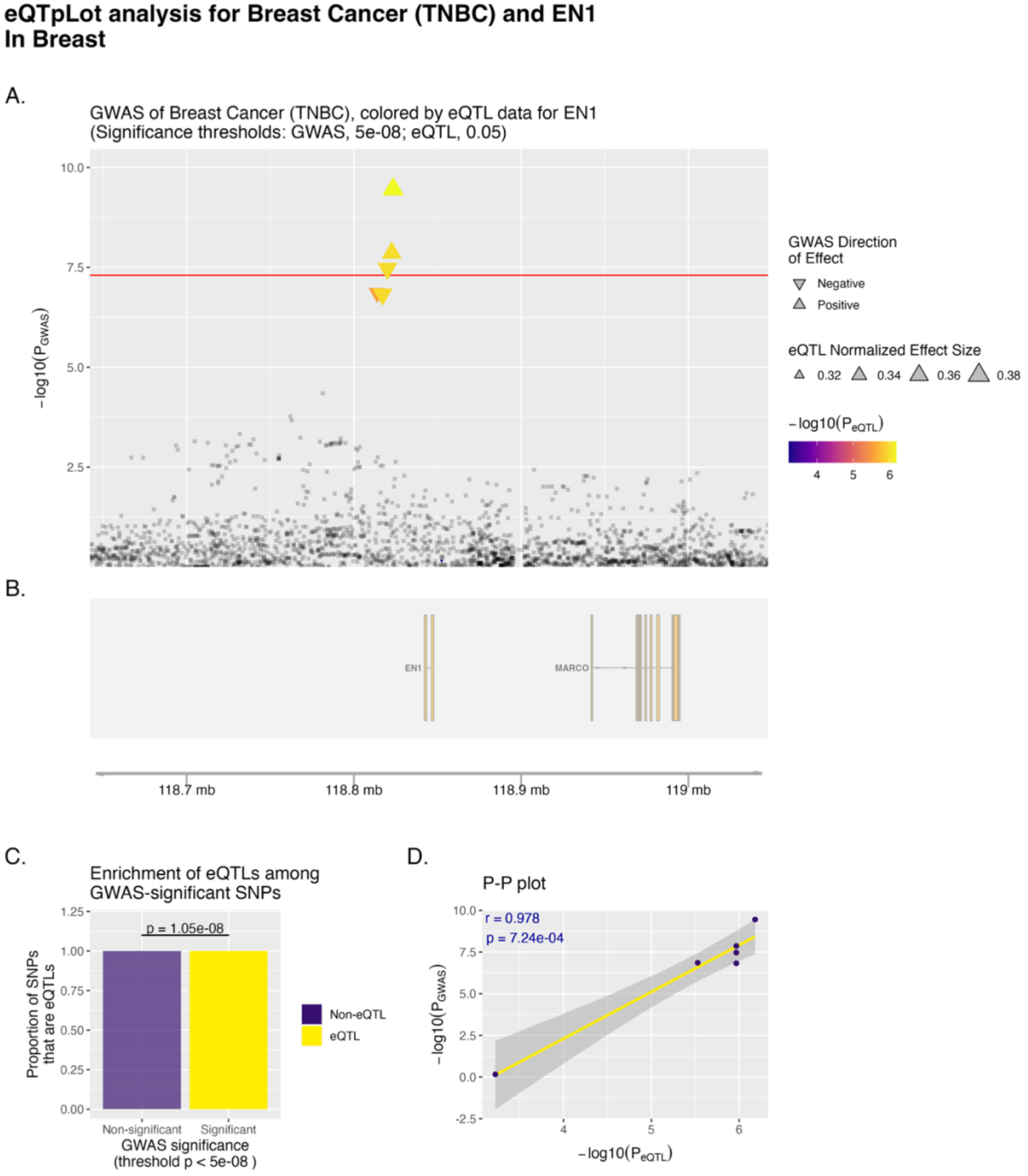

Figure S6. eQTpLot for gene LINC01956 with TNBC breast cancer risk from Exp-TWAS. eQTpLot plots show the colocalization between eQTLs for gene LINC01956 and GWAS signals for TNBC breast cancer risk. **A:** Locus with LINC01956 gene. Points show variant p-values for TNBC breast cancer risk (vertical axis) and LINC01956 expression (color scale). Triangles indicate GWAS effect direction and eQTL effect size. The genome-wide significance threshold (5×10^{-8}) is shown as a red line. **B:** Genomic positions of all genes within the locus. **C:** Enrichment of LINC01956 eQTLs among GWAS-significant variants. **D:** Correlation between P_{GWAS} and P_{eQTL} for LINC01956 and TNBC breast cancer risk, with the computed Pearson correlation coefficient (r) and p-value (p) displayed on the plot.

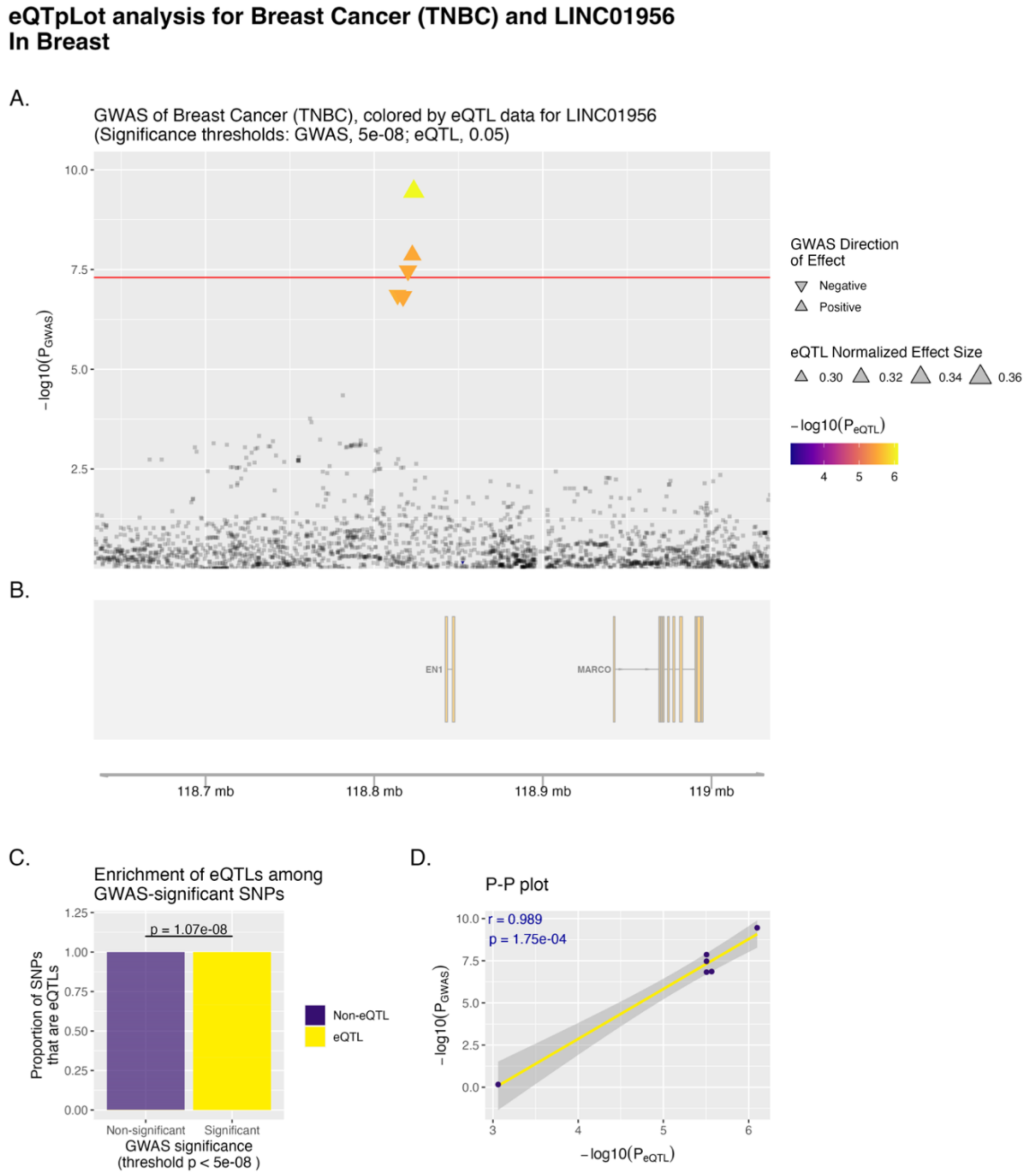

Figure S7. eQTpLot for gene MRPL34 with TNBC breast cancer risk from Exp-TWAS. eQTpLot plots show the colocalization between eQTLs for gene MRPL34 and GWAS signals for TNBC breast cancer risk. **A:** Locus with MRPL34 gene. Points show variant p-values for TNBC breast cancer risk (vertical axis) and MRPL34 expression (color scale). Triangles indicate GWAS effect direction and eQTL effect size. The genome-wide significance threshold (5×10^{-8}) is shown as a red line. **B:** Genomic positions of all genes within the locus. **C:** Enrichment of MRPL34 eQTLs among GWAS-significant variants. **D:** Correlation between P_{GWAS} and P_{eQTL} for MRPL34 and TNBC breast cancer risk, with the computed Pearson correlation coefficient (r) and p-value (p) displayed on the plot.

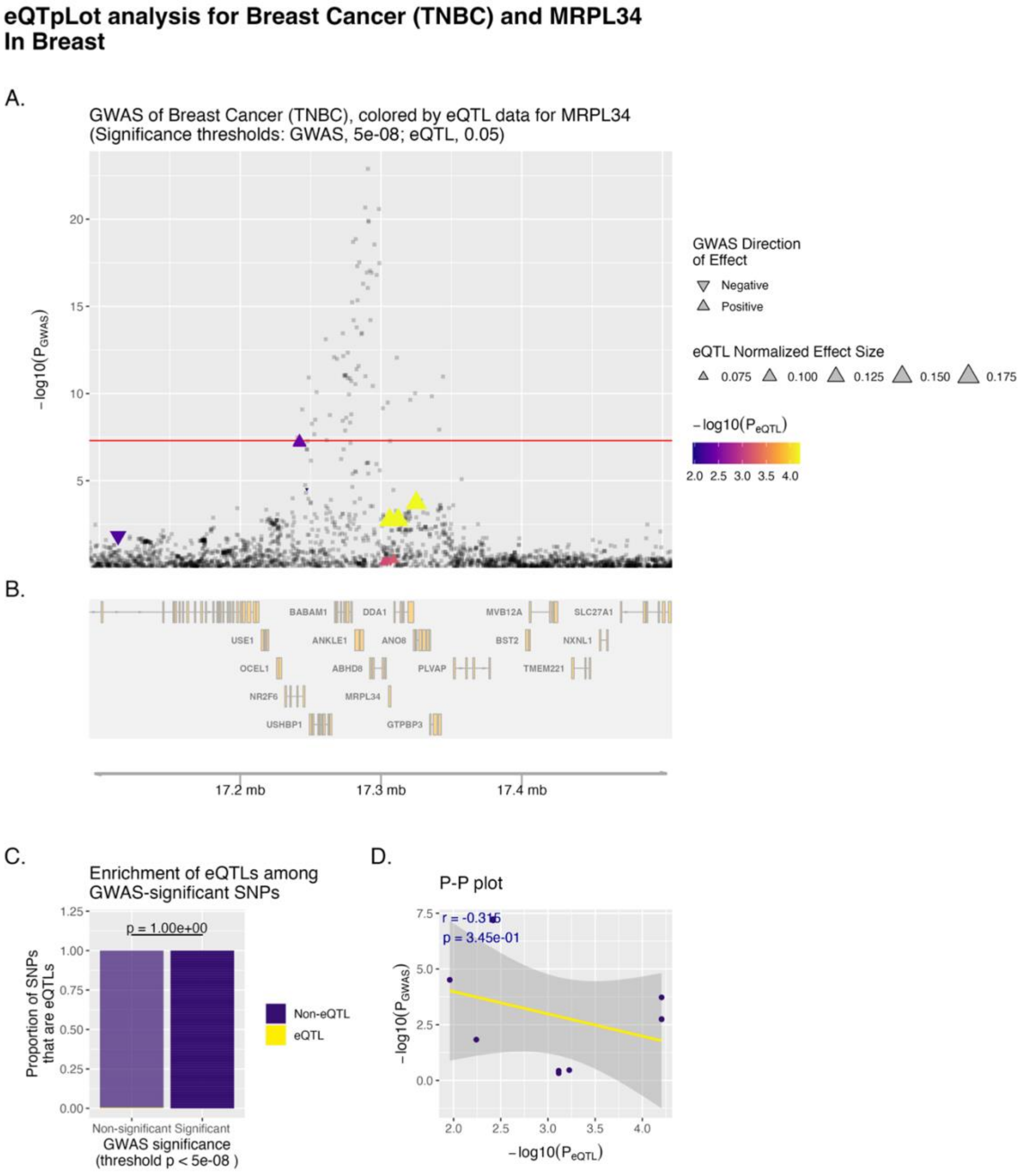

Figure S8. eQTpLot for gene TET2 with overall breast cancer risk from APA-WAS. eQTpLot plots show the colocalization between eQTLs for gene TET2 and GWAS signals for overall breast cancer risk. **A**: Locus with TET2 gene. Points show variant p-values for overall breast cancer risk (vertical axis) and TET2 expression (color scale). Triangles indicate GWAS effect direction and eQTL effect size. **B:** Genomic positions of all genes within the locus. **C:** Enrichment of TET2 eQTLs among GWAS-significant variants. **D:** Correlation between P_{GWAS} and P_{eQTL} for TET2 and overall breast cancer risk, with the computed Pearson correlation coefficient (r) and p-value (p) displayed on the plot.

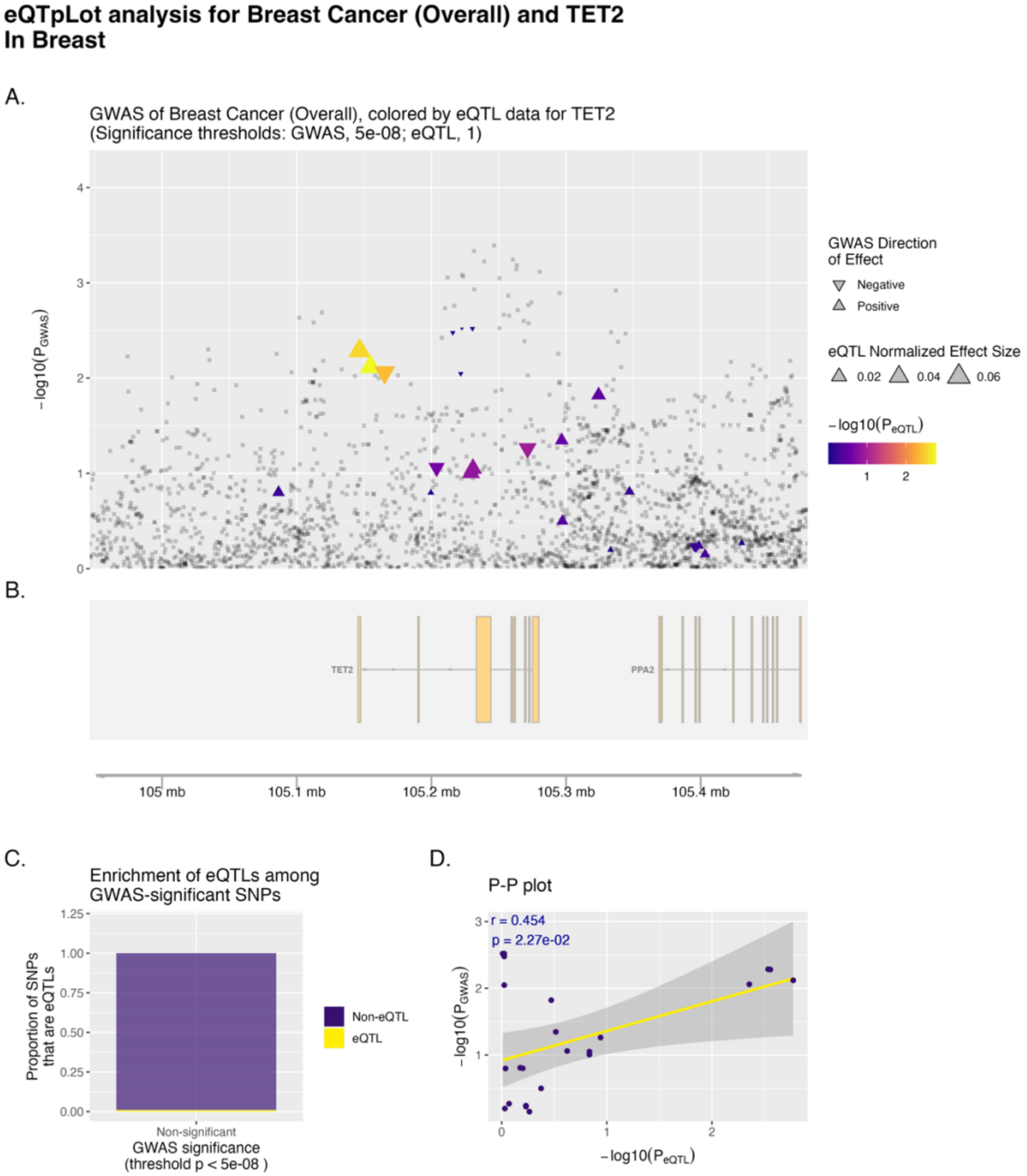

Figure S9. eQTpLot for gene TET2 with ER-negative breast cancer risk from APA-WAS. eQTpLot plots show the colocalization between eQTLs for gene TET2 and GWAS signals for ER-negative breast cancer risk. **A**: Locus with TET2 gene. Points show variant p-values for ER-negative breast cancer risk (vertical axis) and TET2 expression (color scale). Triangles indicate GWAS effect direction and eQTL effect size. **B:** Genomic positions of all genes within the locus. **C:** Enrichment of TET2 eQTLs among GWAS-significant variants. **D:** Correlation between P_{GWAS} and P_{eQTL} for TET2 and ER-negative breast cancer risk, with the computed Pearson correlation coefficient (r) and p-value (p) displayed on the plot.

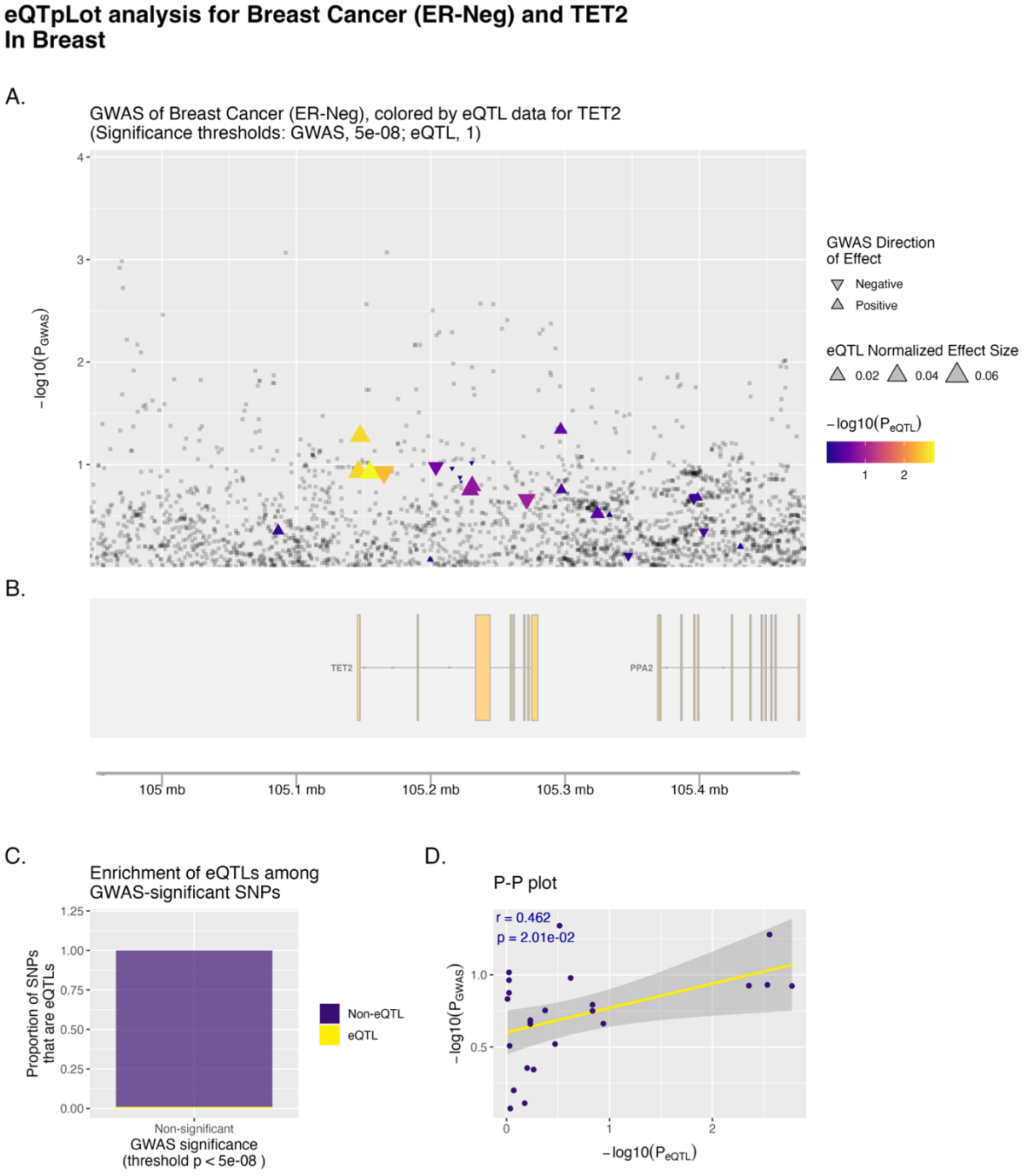

Figure S10. eQTpLot for gene BRD9 with overall breast cancer risk from spTWAS. eQTpLot plots show the colocalization between eQTLs for gene BRD9 and GWAS signals for overall breast cancer risk. **A**: Locus with BRD9 gene. Points show variant p-values for overall breast cancer risk (vertical axis) and BRD9 expression (color scale). Triangles indicate GWAS effect direction and eQTL effect size. The genome-wide significance threshold (5×10^{-8}) is shown as a red line. **B:** Genomic positions of all genes within the locus. **C:** Enrichment of BRD9 eQTLs among GWAS-significant variants. **D:** Correlation between P_{GWAS} and P_{eQTL} for BRD9 and overall breast cancer risk, with the computed Pearson correlation coefficient (r) and p-value (p) displayed on the plot.

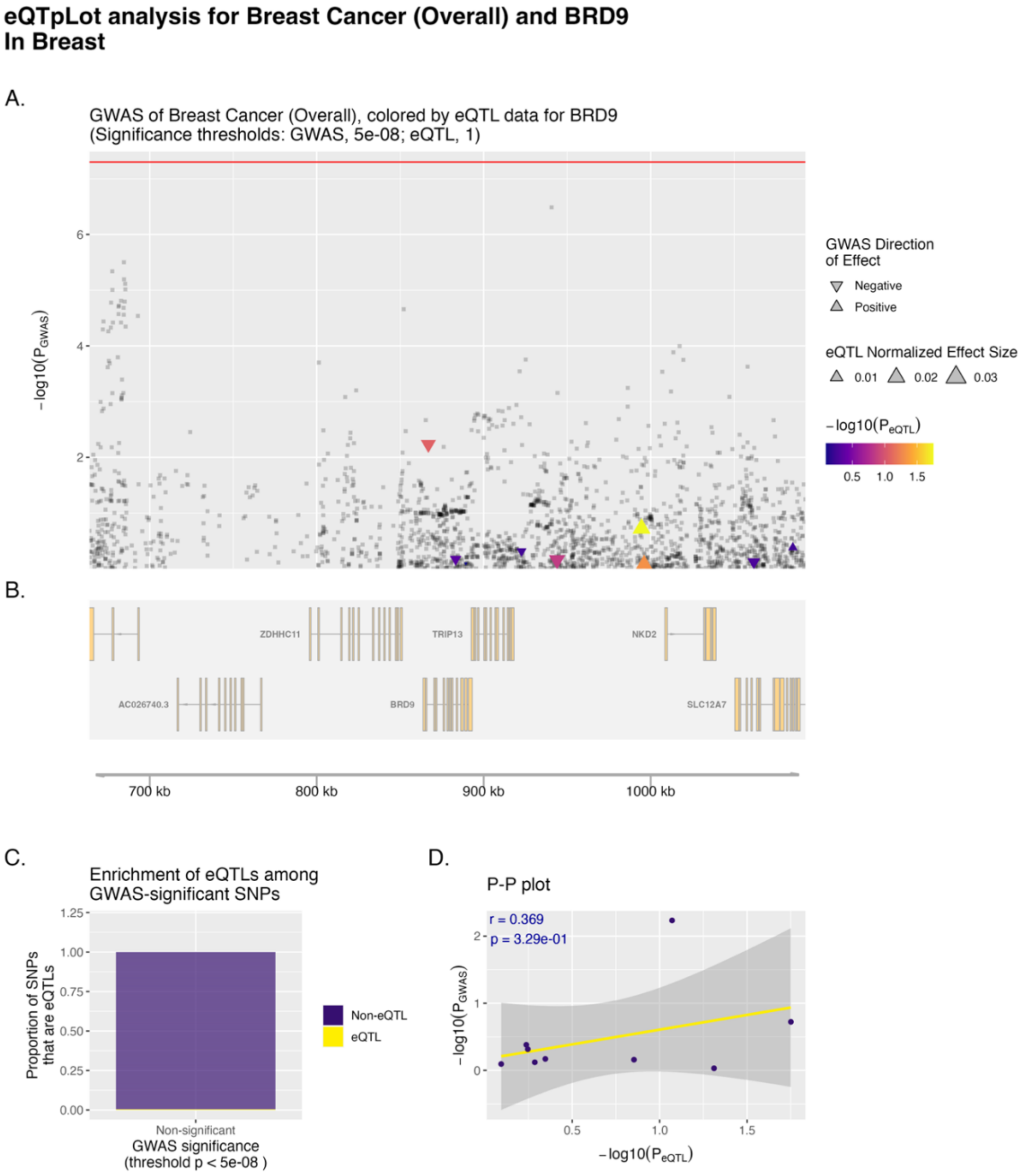

Figure S11. eQTpLot for gene NUP210L with overall breast cancer risk from spTWAS. eQTpLot plots show the colocalization between eQTLs for gene NUP210L and GWAS signals for overall breast cancer risk. **A**: Locus with NUP210L gene. Points show variant p-values for overall breast cancer risk (vertical axis) and NUP210L expression (color scale). Triangles indicate GWAS effect direction and eQTL effect size. **B:** Genomic positions of all genes within the locus. **C:** Enrichment of NUP210L eQTLs among GWAS-significant variants. **D:** Correlation between P_{GWAS} and P_{eQTL} for NUP210L and overall breast cancer risk, with the computed Pearson correlation coefficient (r) and p-value (p) displayed on the plot.

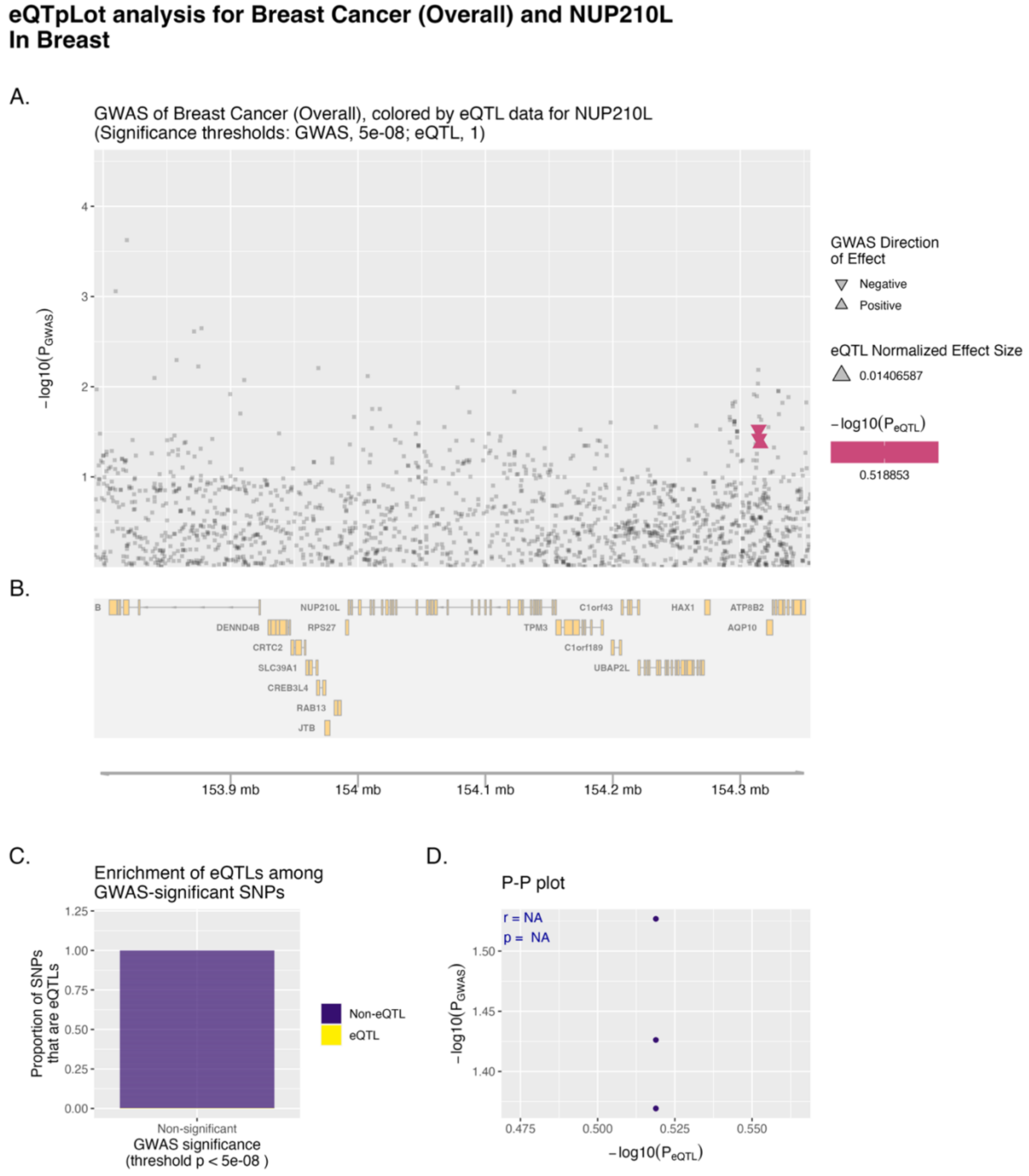

Figure S12. eQTpLot for gene BRD9 with ER-negative breast cancer risk from spTWAS. eQTpLot plots show the colocalization between eQTLs for gene BRD9 and GWAS signals for ER-negative breast cancer risk. **A**: Locus with BRD9 gene. Points show variant p-values for ER-negative breast cancer risk (vertical axis) and BRD9 expression (color scale). Triangles indicate GWAS effect direction and eQTL effect size. The genome-wide significance threshold (5×10^{-8}) is shown as a red line. **B:** Genomic positions of all genes within the locus. **C:** Enrichment of BRD9 eQTLs among GWAS-significant variants. **D:** Correlation between P_{GWAS} and P_{eQTL} for BRD9 and ER-negative breast cancer risk, with the computed Pearson correlation coefficient (r) and p-value (p) displayed on the plot.

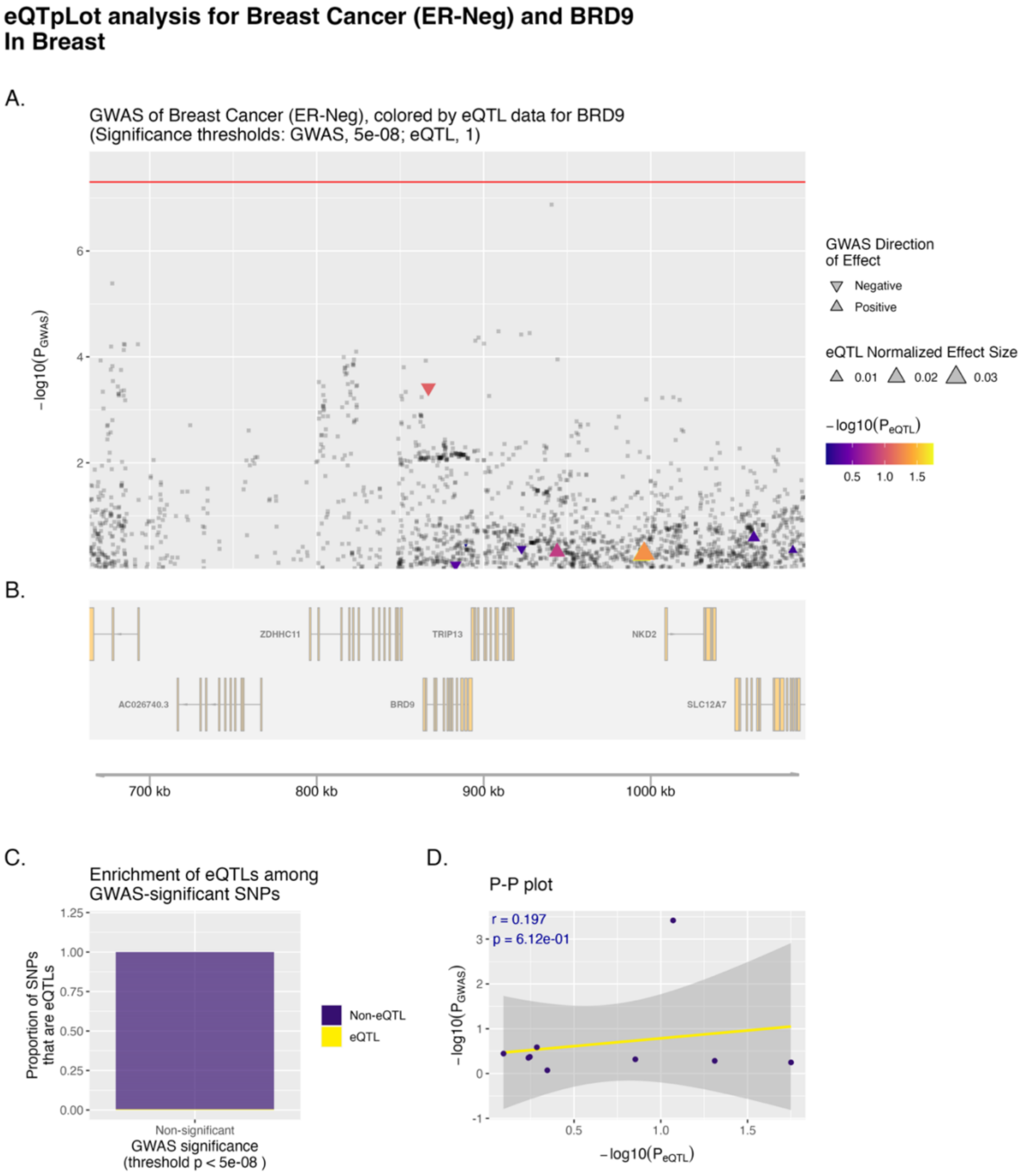

Figure S13. eQTpLot for gene BRD9 with TNBC breast cancer risk from spTWAS. eQTpLot plots show the colocalization between eQTLs for gene BRD9 and GWAS signals for TNBC breast cancer risk. **A**: Locus with BRD9 gene. Points show variant p-values for TNBC breast cancer risk (vertical axis) and BRD9 expression (color scale). Triangles indicate GWAS effect direction and eQTL effect size. The genome-wide significance threshold (5×10^{-8}) is shown as a red line. **B:** Genomic positions of all genes within the locus. **C:** Enrichment of BRD9 eQTLs among GWAS-significant variants. **D:** Correlation between P_{GWAS} and P_{eQTL} for BRD9 and TNBC breast cancer risk, with the computed Pearson correlation coefficient (r) and p-value (p) displayed on the plot.

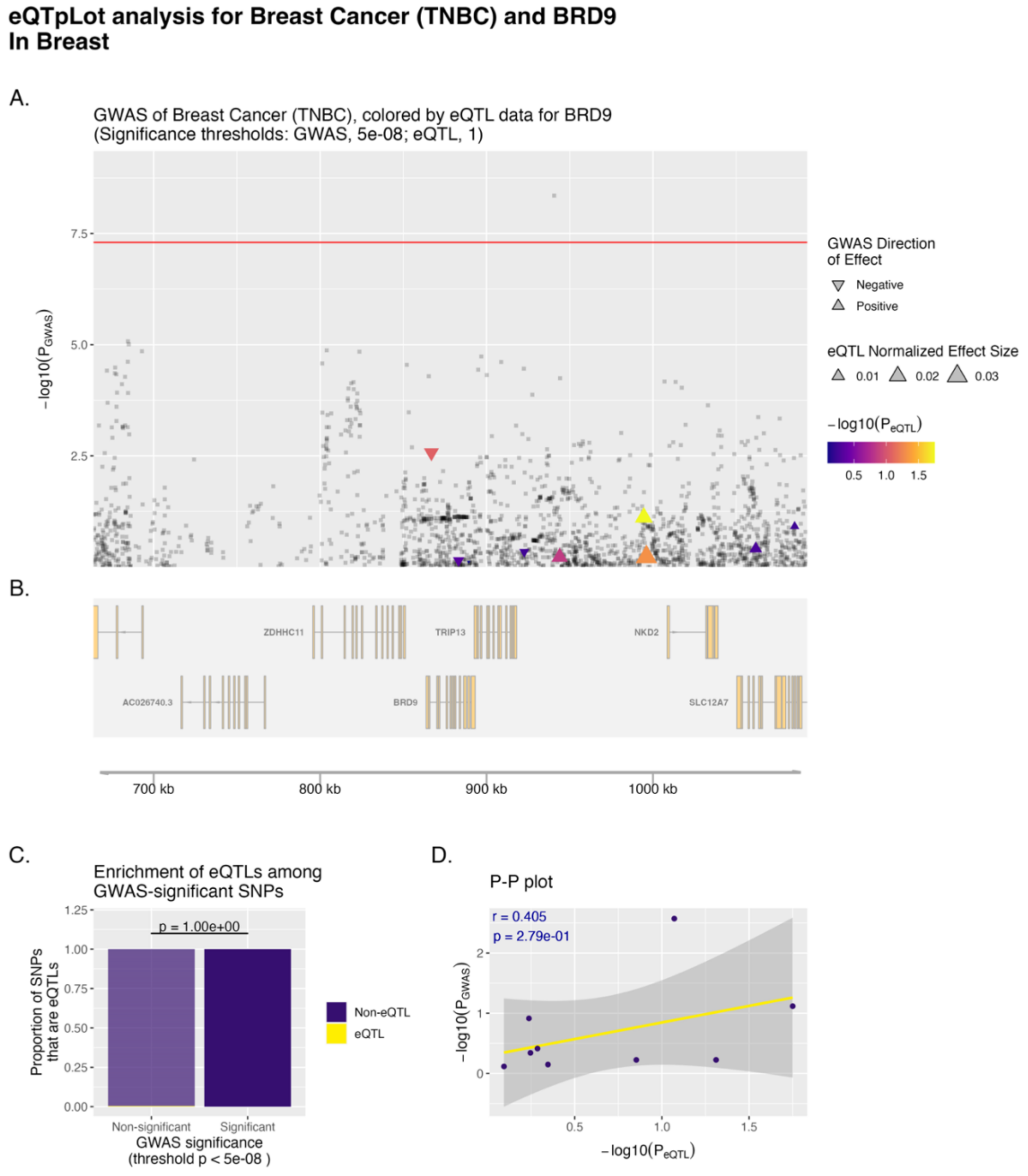

Supplement: Supplementary file 1 — Supplementary Information [file 41467_2024_47650_MOESM1_ESM.pdf]
