# Supplementary material for: Using genome and transcriptome data from African-ancestry female participants to identify putative breast cancer susceptibility genes
Source: Nat Commun. 2024 May 2;15:3718. doi: 10.1038/s41467-024-47650-5 (PMC11065893; doi:10.1038/s41467-024-47650-5)
Supplement: Supplementary file 3 — Reporting Summary [file 41467_2024_47650_MOESM3_ESM.pdf]

nature portfolio

Corresponding author(s): Wei Zheng

Last updated by author(s): Mar 7, 2024

Reporting Summary

Nature Portfolio wishes to improve the reproducibility of the work that we publish. This form provides structure for consistency and transparency in reporting. For further information on Nature Portfolio policies, see our Editorial Policies and the Editorial Policy Checklist.

Statistics

For all statistical analyses, confirm that the following items are present in the figure legend, table legend, main text, or Methods section.

n/a	Confirmed	
☐	☒	The exact sample size (*n*) for each experimental group/condition, given as a discrete number and unit of measurement
☐	☒	A statement on whether measurements were taken from distinct samples or whether the same sample was measured repeatedly
☐	☒	The statistical test(s) used AND whether they are one- or two-sided<br>*Only common tests should be described solely by name; describe more complex techniques in the Methods section.*
☐	☒	A description of all covariates tested
☐	☒	A description of any assumptions or corrections, such as tests of normality and adjustment for multiple comparisons
☐	☒	A full description of the statistical parameters including central tendency (e.g. means) or other basic estimates (e.g. regression coefficient) AND variation (e.g. standard deviation) or associated estimates of uncertainty (e.g. confidence intervals)
☐	☒	For null hypothesis testing, the test statistic (e.g. *F*, *t*, *r*) with confidence intervals, effect sizes, degrees of freedom and *P* value noted<br>*Give P values as exact values whenever suitable.*
☒	☐	For Bayesian analysis, information on the choice of priors and Markov chain Monte Carlo settings
☒	☐	For hierarchical and complex designs, identification of the appropriate level for tests and full reporting of outcomes
☐	☒	Estimates of effect sizes (e.g. Cohen's *d*, Pearson's *r*), indicating how they were calculated

Our web collection on statistics for biologists contains articles on many of the points above.

Software and code

Policy information about availability of computer code

Data collection	No software was used for data collection.
Data analysis	Plink v1.9 was used to analyze the genotype data. A two-pass method of the Spliced Transcripts Alignment to a Reference (STAR) software was used for raw data alignment to the human reference genome (hg38). Gene expression levels were quantified from aligned BAM files using RNAseQC v1.1.9. Probabilistic estimation of expression residuals (PEER) factors were calculated to correct for batch effects and other potential experimental confounders in further model building. We quantified levels of APA events by using the Percentage of Distal polyA site Usage Index (PDUI) estimated from DaPars v2.0 for each sample from aligned RNA-Seq data. We estimated exon junction levels from aligned RNA-Seq data by using a probabilistic framework, MISO (Mixture-of-Isoforms). Genetic prediction models for each type of transcriptomic events were built using the elastic net model implemented in the glmnet R package. Based on the weight matrix and the summary statistics data on SNPs from GWAS data, we evaluated the association between genetically predicted transcriptome levels and breast cancer risk using the method from the S-PrediXcan tool. GCTA-COJO analyses were performed to calculate the statistical significance with cancer risk after adjusting for the nearest lead variant. All statistical analyses were performed using R v4.0.5.

For manuscripts utilizing custom algorithms or software that are central to the research but not yet described in published literature, software must be made available to editors and reviewers. We strongly encourage code deposition in a community repository (e.g. GitHub). See the Nature Portfolio guidelines for submitting code & software for further information.

Data

Policy information about availability of data

All manuscripts must include a data availability statement. This statement should provide the following information, where applicable:

- Accession codes, unique identifiers, or web links for publicly available datasets
- A description of any restrictions on data availability
- For clinical datasets or third party data, please ensure that the statement adheres to our policy

The genotyping and RNA-Seq data of samples from the Susan G. Komen Tissue Bank generated in this study have been deposited in the dbGaP database under accession code phs003535.v1.p1 [https://www.ncbi.nlm.nih.gov/projects/gap/cgi-bin/study.cgi?study_id=phs003535.v1.p1]. Summary-level statistics data for the AABCG study are available at the GWAS Catalog under accession number GCST90296719 for overall breast cancer, GCST90296720 for ER-positive breast cancer, GCST90296721 for ER-negative breast cancer, and GCST90296722 for TNBC. GENCODE datasets are available fromhttps://www.gencodegenes.org/human/release_26.html.

Research involving human participants, their data, or biological material

Policy information about studies with human participants or human data. See also policy information about sex, gender (identity/presentation), and sexual orientation and race, ethnicity and racism.

Reporting on sex and gender	Only samples from women were used in this study and the findings will be applied to women only.
Reporting on race, ethnicity, or other socially relevant groupings	Only samples from African-ancestry women were used in this study and race was not used in the analysis.
Population characteristics	African-ancestry women recruited from studies conducted in the U.S. and Africa
Recruitment	All study participants were recruited from previous studies.
Ethics oversight	The study has been approved by the Vanderbilt University Medical Center institutional review board

Note that full information on the approval of the study protocol must also be provided in the manuscript.

Field-specific reporting

Please select the one below that is the best fit for your research. If you are not sure, read the appropriate sections before making your selection.

☒ Life sciences ☐ Behavioural & social sciences ☐ Ecological, evolutionary & environmental sciences

For a reference copy of the document with all sections, see nature.com/documents/nr-reporting-summary-flat.pdf

Life sciences study design

All studies must disclose on these points even when the disclosure is negative.

Sample size	The study includes 150 women who provided normal breast tissue samples and approximately 40,000 breast cancer cases and controls who were included in the African-ancestry Breast Cancer Genetic Study.
Data exclusions	Samples that failed in quality control were excluded.
Replication	No replication was conducted. However, Bonferroni correction was used to minimize type 1 error due to multiple comparisons.
Randomization	This TWAS used statistical models to test the associations between genetically predicted transcriptomic events and breast cancer risk. Since the analysis is based on preexisting genetic variation, no randomization is applicable.
Blinding	The “exposure” (gene expression levels) and the “outcomes” (breast cancer risk) in TWAS are determined from using data from normal breast tissue samples and data from the AABCG, respectively. These variables are not through administering treatments, therefore blinding in the traditional sense does not apply.

Reporting for specific materials, systems and methods

We require information from authors about some types of materials, experimental systems and methods used in many studies. Here, indicate whether each material, system or method listed is relevant to your study. If you are not sure if a list item applies to your research, read the appropriate section before selecting a response.

Materials & experimental systems

n/a	Involved in the study
☒	☐ Antibodies
☒	☐ Eukaryotic cell lines
☒	☐ Palaeontology and archaeology
☒	☐ Animals and other organisms
☒	☐ Clinical data
☒	☐ Dual use research of concern
☒	☐ Plants

Methods

n/a	Involved in the study
☒	☐ ChIP-seq
☒	☐ Flow cytometry
☒	☐ MRI-based neuroimaging

Plants

Seed stocks

Report on the source of all seed stocks or other plant material used. If applicable, state the seed stock centre and catalogue number. If plant specimens were collected from the field, describe the collection location, date and sampling procedures.

Novel plant genotypes

Describe the methods by which all novel plant genotypes were produced. This includes those generated by transgenic approaches, gene editing, chemical/radiation-based mutagenesis and hybridization. For transgenic lines, describe the transformation method, the number of independent lines analyzed and the generation upon which experiments were performed. For gene-edited lines, describe the editor used, the endogenous sequence targeted for editing, the targeting guide RNA sequence (if applicable) and how the editor was applied.

Authentication

Describe any authentication procedures for each seed stock used or novel genotype generated. Describe any experiments used to assess the effect of a mutation and, where applicable, how potential secondary effects (e.g. second site T-DNA insertions, mosiacism, off-target gene editing) were examined.
